# Experimental and Theoretical Perspectives of the Noyori-Ikariya Asymmetric Transfer Hydrogenation of Imines

**DOI:** 10.3390/molecules19066987

**Published:** 2014-05-28

**Authors:** Jiří Václavík, Petr Šot, Jan Pecháček, Beáta Vilhanová, Ondřej Matuška, Marek Kuzma, Petr Kačer

**Affiliations:** 1 Department of Organic Technology, Institute of Chemical Technology, Technická 5, CZ-166 28 Prague, Czech Republic; 2 Laboratory of Molecular Structure Characterization, Institute of Microbiology, v.v.i., Academy of Sciences of the Czech Republic, Vídeňská 1083, CZ-142 20 Prague, Czech Republic

**Keywords:** ruthenium, asymmetric hydrogenation, imine, ligand, sulfonyl, arene, DFT

## Abstract

The asymmetric transfer hydrogenation (ATH) of imines catalyzed by the Noyori-Ikariya [RuCl(*η*^6^-arene)(*N*-arylsulfonyl-DPEN)] (DPEN = 1,2-diphenylethylene-1,2-diamine) half-sandwich complexes is a research topic that is still being intensively developed. This article focuses on selected aspects of this catalytic system. First, a great deal of attention is devoted to the *N*-arylsulfonyl moiety of the catalysts in terms of its interaction with protonated imines (substrates) and amines (components of the hydrogen-donor mixture). The second part is oriented toward the role of the *η*^6^-coordinated arene. The final part concerns the imine substrate structural modifications and their importance in connection with ATH. Throughout the text, the summary of known findings is complemented with newly-presented ones, which have been approached both experimentally and computationally.

## 1. Introduction

**Scheme 1:**
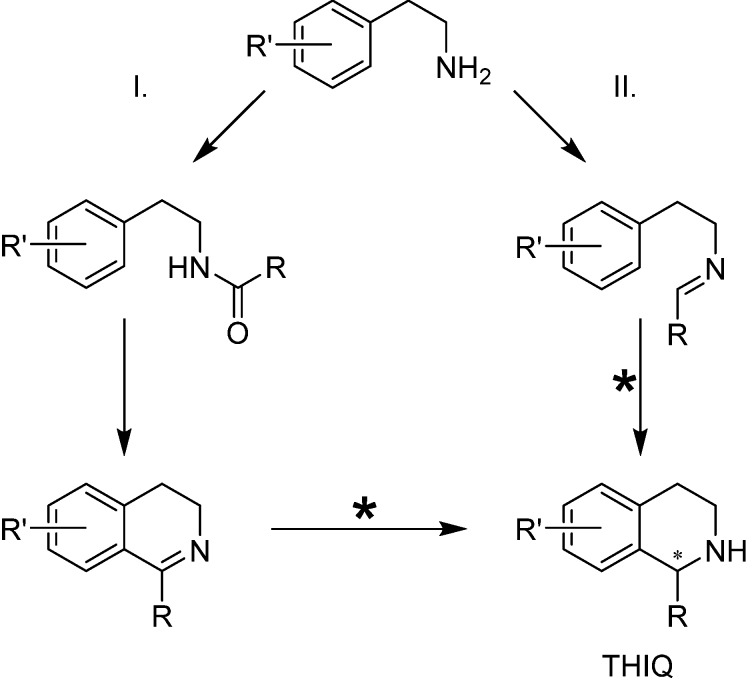
Typical routes towards the preparation of 1,2,3,4-tetrahydroisoquinolines (THIQs). Route I proceeds via the Schotten-Baumann acylation of the starting amine, Bischler-Napieralski cyclization of the resulting amide, and reduction of the C=N bond. Route II comprises the formation of an imine which subsequently undergoes the Pictet-Spengler reaction. The steps in which asymmetry originates are marked with asterisks.

The effective synthesis of molecules based on the 1,2,3,4-tetrahydroisoquinoline (THIQ) fragment (Scheme 1) remains a significant point of interest because these substances quite often exhibit bioactivity, thus having a great potential as drugs [1]. The main routes toward THIQs include the Pictet-Spengler reaction [2] and hydrogenation of dihydroisoquinolines (DHIQs). DHIQs are typically synthesized via the Bischler-Napieralski cyclodehydration [3], preceded by the acylation of the corresponding amine under the Schotten-Baumann conditions.

Bioactive THIQ molecules are very often chiral, which implies that usually only one enantiomer is desired and efficient methods of synthesis of enantiopure THIQs are sought-after. As both Pictet-Spengler reaction and hydrogenation of DHIQs lead to racemic mixtures of product isomers, these need to be further separated in case a particular enantiomer is needed. A recent review by Lorenz and Seidel-Morgenstern provides an excellent overview of the techniques used for chiral resolution [4].

Asymmetric synthesis now belongs to the main directions in modern organic chemistry [5,6]. The optically enriched products can be obtained directly from prochiral precursors and such reactions are often feasible under mild conditions. They are atom-economic, contrary to the chiral resolution techniques that are associated with large amounts of waste. Both imine hydrogenation and the Pictet-Spengler reaction [7] can nowadays be performed in an asymmetric fashion. Since this paper is exclusively focused on hydrogenation, the latter will be omitted in the following text.

The asymmetric transfer hydrogenation (ATH) of imines catalyzed by [RuCl(*η*^6^-arene)(*N*-arylsulfonyl-DPEN)] (DPEN = 1,2-diphenylethylene-1,2-diamine) half-sandwich complexes (Scheme 1) [8] is currently a well-established, yet still intensively studied method for the synthesis of enantioenriched amines. We have summarized the approaches addressing the ecological aspects of the reaction by using water or ionic liquids as solvents and recycling of the catalyst (typically by two-phase systems or its immobilization onto solid supports) [9]. Many active pharmaceutical ingredients (APIs) or their precursors have been prepared by employing this procedure in some of its numerous modifications that have been reported over the years [10].

Every successful application of a chemical reaction is preceded by detailed and extensive studies on the fundamental level. ATH is not an exception. A typical ATH system consists of a catalyst, substrate, hydrogen donor and solvent (though solvent-free conditions have been studied as well, e.g., in [11,12]). Obvious options for the optimization of an ATH reaction for a particular substrate thus involve the choice of the appropriate organometallic complex, solvent and hydrogen donor (usually the HCOOH/triethylamine mixture [13,14], or sodium formate when performing the reaction in water [15], but other donors can be used such as propan-2-ol [16], methanol and ethanol [17], or combinations thereof [18]). However, each of those components can be fine-tuned separately owing to the vast number of their modifications available, which dramatically increases the total amount of potential combinations. Trial-and-error experimental screening is both time-consuming and costly, so it is very convenient to have as much systematic research outcomes as possible, which can help narrowing down the alternatives eventually taken into consideration.

In general, basic research of ATH can be carried out in two directions – experimental and theoretical. The tangible results of experimental work usually provide us with actual, “real” information about the studied catalytic system (in case no systematic error occurs). The scope of available methods for the investigation of the catalytic system is quite broad, but not infinite, so every researcher stumbles upon certain limits at some point. Moreover, the years invested into the examination of the ATH reaction mechanism have shown its considerable complexity. The experimental observations are thus very often manifested as “averaged” views of the catalytic system, where we are unable to analyze each component separately. 

Computational chemistry provides us with a variety of powerful tools allowing us to explore otherwise hardly accessible properties of molecular systems such as the geometry of transition states, energy differences of reaction intermediates, steric aspects, *etc.* However, the accuracy of these computations is limited by the fact that they can be extremely hardware-demanding, so one must at first try to balance the accuracy and computational cost according to the desired properties and precision. It is thus recommended to exploit both the experimental and theoretical techniques for the investigation of the catalytic system. The computations should be related to existing experimental data and, *vice versa*, the experiments can be complemented with carefully designed *in silico* studies. This work concerns selected individual aspects of the ATH system by employing both views discussed above.

## 2. The Ru^II^ Catalytic Complex

### 2.1. The N-R-Sulfonyl Fragment

**Figure 1:**
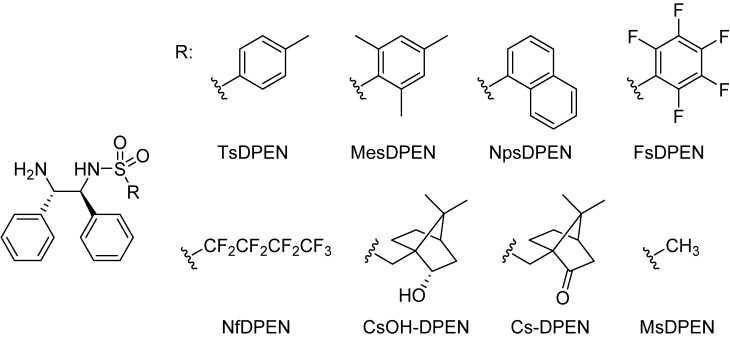
Selected structures of *N*-aryl and *N*-alkylsulfonyl-DPEN ligands that have been reported.

The *N*-R-sulfonyl fragment has been subject to a number of structural modifications (Figure 1). Noyori *et al.* originally published catalysts with various *N*-aryl- or *N*-alkylsulfonyl moieties of the TsDPEN ligand, such as mesityl (Mes), tosyl (Ts), 1-naphthyl (Np) and trifluoromethyl [13,16]. Later, the methanesulfonyl-DPEN (MsDPEN) ligand was introduced and proved effective in the hydrogenation of ketones and imines using molecular hydrogen [19,20]. *N*-(Borneol-10-sulfonyl)-DPEN (CsOH-DPEN) and *N*-(camphor-10-sulfonyl)-DPEN (Cs-DPEN) are among other popular ligands that were employed in the hydrogenation of both ketones [21,22,23,24] and imines [25]. Mohar * et al.* reported the perfluorinated FsDPEN and NfDPEN ligands [26] which they used for the ATH of a racemic chiral *β*-hydroxy-*α*-amino acid via dynamic kinetic resolution. They measured the p*K*_a_ values of the ligands used in their work by potentiometric titration and they found that the highest catalytic activity was related to the lowest p*K*_a_ values and *vice versa*. Later they reported a series of *N*-(*N*,*N*-dialkylamino)sulfamoyl-DPEN [27,28], nitro-, chloro, methoxy- and isopropyl-substituted (later denoted TIPPS-DPEN [29]) PhSO_2_-DPEN ligands [30] for the ATH of ketones. Wills * et al.* further extended the spectrum of *N*-arylsulfonyl moieties by incorporating alkyl, fluorine and methoxy substitutions to the phenyl ring [31].

More profound modifications of the *N*-R-sulfonyl fragment have been shown, aiming at substantial changes in catalyst’s properties, e.g., for immobilization, or for conducting ATH in different media (water, ionic liquids). This has been reviewed previously [10].

It is evident that the literature contains many interesting examples of structural variations. However, the scope of available ligands is difficult to compare due to inconsistent reaction conditions. So far it seems that every modification finds its application with certain substrates, which can be reached by experimental screening. In order to shed some light upon the importance of the sulfonyl fragment itself from the mechanistic point of view, we present below a concise summary of our findings in this regard.

### 2.2. The Sulfonamide Moiety

#### 2.2.1. Interaction of the *N*-R-Sulfonyl Moiety with Imine Substrates

**Figure 2:**
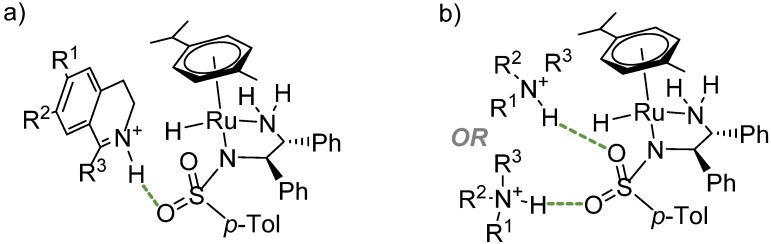
(**a**) Interaction of the sulfonyl group with protonated DHIQ via a hydrogen bond (green dashed line). (**b**) Interaction of the sulfonyl group with protonated amines viahydrogen bonds (green dashed line).

It is assumed that imine substrates enter the catalytic cycle in the iminium form [32,33]. This fact is closely related to our gas-phase computational study in which we suggested an explanation of the importance of the sulfonyl group [34]. As shown in Figure 2a, the protonated substrate can form a hydrogen bond with the sulfonyl group, which represents the first step of the hydrogen transfer mechanism. The work in detail describes that a different orientation of the substrate molecule would afford the wrong enantiomer of the product (*i.e.*, opposite than the one observed experimentally). 

Ward *et al.* have recently reported a computational study on the mechanism of imine reduction using their artificial hydrogenases [35]. The enzymes are composed of Ir(III) complexes bearing biotin ligands, which serve as cofactors of streptavidin. Their study confirmed the preference of the “ionic” transition states in which the proton and hydride are transferred asynchronously. However, according to their findings, there is a sound possibility that the imine substrate approaches the catalyst differently, *i.e.*, by forming a C=N^+^–H∙∙∙NH_2_- hydrogen bond with the NH_2_ group of the diamine ligand. In fact, this path was energetically more favourable than the one via the C=N^+^–H∙∙∙O=SO- bond as suggested by us (Figure 2a). The authors comment that the difference might be due to the usage of different DFT functionals and solvation models, or might be caused by the difference between the catalysts studied. However, both studies have shown that the activation of imines (in this case via protonation) is important for the ATH reaction. This issue is further discussed below in Section 3.1

#### 2.2.2. Interaction of the *N*-R-Sulfonyl Moiety with Nitrogenous Bases Present in the Hydrogen Donor Mixture

We have shown in the previous section that the sulfonyl group can form hydrogen bonds with protonated imines. In the course of our investigation of the reaction mechanism, we came to a similar conclusion by employing spectroscopic techniques [36]. When the HCOOH/triethylamine (5/2 molar ratio) hydrogen donor mixture is used for hydrogenation, triethylamine is protonated under such conditions [37] and therefore it is likely to bond to the sulfonyl group as well. When we measured the mixture of catalyst [RuCl(*η*^6^-*p*-cymene)TsDPEN], formic acid and tertiary nitrogenous base (triethylamine, diisopropylethylamine and 1,4-diazabicyclo[2.2.2]octane) in acetonitrile, the ion clusters observed corresponded to the Ru^II^ complex and base. The molecular system was subsequently measured by vibrational circular dichroism (VCD) that suggested a hydrogen bond of the N–H∙∙∙O=S type between the catalyst and the base (depicted in Figure 2b).

The expected supramolecular associate was also studied by NMR spectroscopy. Our first intentions were based on the measurement of the Nuclear Overhauser Effect (NOE) and diffusion ordered spectroscopy (DOSY) experiments. However, such measurements were hampered particularly by the strong excess of the HCOOH-base mixture over the catalyst and continuous and multiple chemical reactions occurring in the mixture, which disallowed us to perform longer experiments [38]. Hence, we turned our attention towards the amine molecule. The natural abundance of the ^15^N nitrogen nucleus, the only stable isotope with the nuclear spin of –1/2, is as low as 0.35%, and thus it was practically impossible to perform any NMR experiments (with the aim of detecting minor species) focused on the nitrogen nucleus with amines that are commonly available.

**Figure 3:**
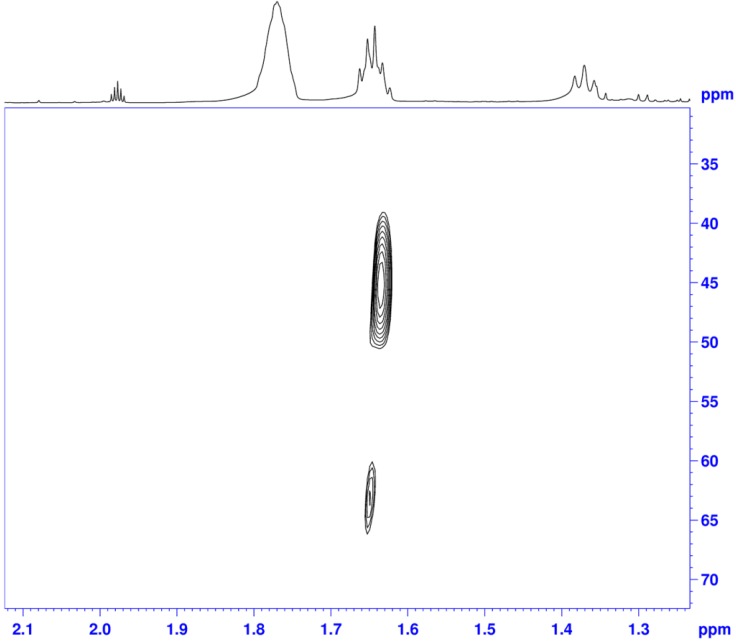
A section of a ^1^H-^15^N gHMBC NMR spectrum of a mixture of catalyst [RuCl(*η*^6^-*p*-cymene)TsDPEN], HCOOH, piperidine-^15^N and acetonitrile-*d*_3_[36].

Hence, we prepared piperidine-^15^N from commercially available pyridine-^15^N (Section 1 in Supplementary materials [38]). Catalyst [RuCl(*η*^6^-*p*-cymene)TsDPEN] was then mixed with piperidine-^15^N (10 eq to catalyst), HCOOH (2.5 eq to piperidine) and acetonitrile-*d*_3_[38]. The excess of the HCOOH/piperidine mixture was based on rather low solubility of the catalyst and our aim to observe the ^15^N-labelled compound, which required its higher quantity. A ^1^H–^15^N gHMBC spectrum was acquired. Piperidine typically resonates at 30–40 ppm (depending on the solvent) in a ^15^N-NMR spectrum [39,40], but the chemical shift can increase by approximately 10 ppm through protonation [41]. Surprisingly, two different crosspeaks were observed in the F1 dimension at 45 and 64 ppm (Figure 3). While the major peak corresponds to protonated piperidine, the other one must represent different piperidine molecule given that no other ^15^N-labelled compound was present in the mixture. This observation can be interpreted in the same fashion like the conclusions drawn above: a certain amount of piperidine is bound to the catalyst molecule.

The sulfonyl-ammonium associates were subjected to DFT calculations in order to provide a theoretical support for the observed interaction of the amine with the ruthenium complex (Table 1; Section 2.1 in Supplementary materials [38,42]). As suggested above, the ability of the *N*-protonated amine molecules to form hydrogen bonds with one of the oxygen atoms of the sulfonamide group [36] was evaluated. The structures corresponding to entries 1–5 (Table 1) were optimized *in vacuo*, after which the MP2 energy calculations were performed on the optimized structures. Similarly, the structures corresponding to entries 6–10 were optimized with solvation effects taken into account (acetonitrile, IEFPCM model). Again, MP2 energy calculations were performed on the optimized structures. The results in vacuum showed the associate with *n*-butylamine (entry 1) to have the strongest hydrogen bond. The other amines (piperidine, triethylamine and tributylamine, entries 2–5) displayed fairly similar bond energies. However, when the solvation model was applied, the comparison of triethylamine (entry 9) and piperidine ([43], entries 7 and 8) revealed significantly lower bond energy in the latter case. The molecule of *n*-butylamine (entry 6) showed bond energy values comparable with those of piperidine. The last amine in the series was tributylamine (entry 5), which displayed the highest bond energy of all examined species. To sum up, the trend was completely opposite when compared with the computations that did not consider solvation. Both species creating the associate are solvated under the reaction conditions. We assume that the molecules of solvent prevent the catalyst and substrate from forming a stronger hydrogen bond, which is the reason for eminently lower absolute values of the bond energies (entries 6–10). However, the real mode of solvation can be strikingly different from the applied theoretical model (IEFPCM in this case), which e.g., cannot reflect the well-known possibility of coordination of acetonitrile to ruthenium. Modelling of such associates is thus essentially dependent on proper selection of the solvation model (*vide infra*).

**Table 1:** Single-point energies and Gibbs free energies of the hydrogen bond between catalyst and protonated amine molecules [38].

Entry	Solvation	Amine	Δ*E*_b_ [kJ·mol^−1^] (MP2//DFT)	Δ*E*_b_ [kJ·mol^−1^] (DFT//DFT)	Δ*G*_b_ [kJ·mol^−1^] (DFT//DFT)
1	none	*n*-Butylamine	–220.0	–234.6	–177.6
2	none	Piperidine	–208.4	–222.0	–166.2
3	none	Piperidine	–204.0	–210.7	–159.1
4	none	Triethylamine	–203.1	–210.0	–157.3
5	none	Tributylamine	–206.8	–210.3	–158.4
6	IEFPCM	*n*-Butylamine	–76.2	–81.4	–24.1
7	IEFPCM	Piperidine	–79.6	–81.5	–25.9
8	IEFPCM	Piperidine	–78.2	–77.2	–26.7
9	IEFPCM	Triethylamine	–89.4	–87.3	–31.0
10	IEFPCM	Tributylamine	–95.5	–93.5	–46.5

We performed the corresponding kinetic experiments (Table 2) in parallel with our calculations. The results did not follow the trends observed in the ATH experiments done earlier by us *in situ* in an NMR tube [36], where triethylamine and sterically more demanding *N*,*N*-diisopropyl(ethyl)amine surpassed other (mostly secondary) amines in reactivity. Here, the reactions were done in round-bottom flasks under previously reported reaction conditions [44]. This observation highlights the reaction’s sensitivity to the proper setup of the reaction conditions. The reaction rates correlated best with the calculations obtained *in vacuo* – *n*-butylamine displayed the lowest reaction rate together with the highest bond energy. The remaining amines showed similar rate and energy values. Hence, it can be seen that the computations considering solvation via the IEFPCM model did not agree with experimental data in this case.

**Table 2:** ATH of 1-methyl-3,4-DHIQ with [RuCl(*η*^6^-*p*-cymene)(*S*,*S*)-TsDPEN] and HCOOH/amine (5/2) hydrogen-donor mixtures ^[a]^. 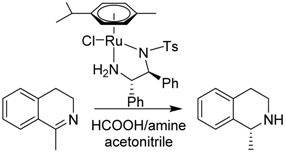

Entry	Amine	Initial reaction rate [mmol∙min^−1^∙mmol_cat_^−1^]	*ee* [%] ^[b]^
1	*n*-Butylamine	1.73	85
2	Piperidine	2.75	85
3	Triethylamine	2.86	83
4	Tributylamine	2.55	84

^[a]^ Initial reaction rates were calculated from the linear part of each conversion curve by the least-squares linear regression method. The reaction conditions were adapted from our previous study [44]. ^[b]^ The *ee* values were determined by employing a GC method reported by us [45].

**Figure 4:**
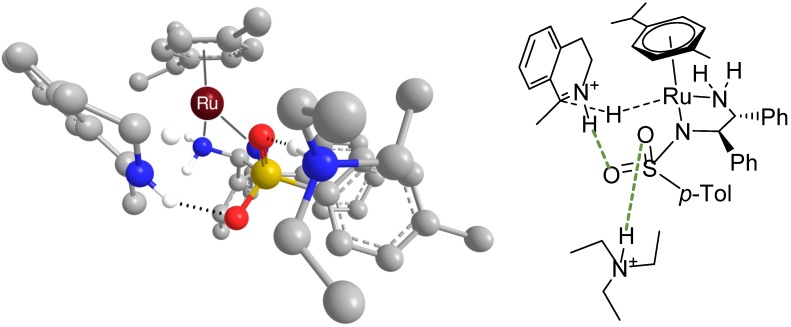
Transition state of hydrogenation of 1-methyl-DHIQ with [RuH(*η*^6^-*p*-cymene)(*R*,*R*)-TsDPEN] with Et_3_NH^+^ hydrogen-bonded to one oxygen atom of the sulfonyl group (optimized geometry).

Nonetheless, in all cases, the bond energies between the catalytic complex and amines were found to be significant and thus they give a theoretical support to the idea of a non-covalent interaction between the catalytic complex and the amine [34]. It is possible that this occurs during the ATH reaction – Figure 4 depicts a calculated transition state of the hydrogenation of 1-methyl-DHIQ with the triethylammonium ion hydrogen-bonded to the sulfonyl group (the computational method was identical to that described in the Supplementary materials, Section 2.1).

### 2.3. The η^6^-Arene Ligand

It is a well-known phenomenon that the enantioselective course of the reaction is a direct result of the energetic difference between the *Re* and *Si* pathways. This difference can be viewed as a multiparametric relation because it includes all stabilizing and destabilizing interactions present within the transition states. Naturally, only those with major contribution to this difference will have a significant impact on the resulting asymmetry. One of these interactions is the CH/*π* weak hydrogen bond (also known as the CH/*π* interaction) which can be formed between the aromatic part of the substrate and the *η*^6^-arene ligand [46]. This interaction offers significant stabilization provided that the substrate molecule adopts specific orientation which permits its formation. A study by Yamakawa and Noyori suggests that this interaction can provide stabilization of up to 12.3 kJ·mol^−1^, which clearly demonstrates its cardinal role [46]. The attractive force is not limited to aromatic substrates – recently, several reports have emerged on the enantioselective hydrogenation of alkynyl ketones, showing that the *π* electrons of alkynes can also interact with the *η*^6^-arene [47,48,49]. The necessity of the aforementioned interaction is also one of several key aspects which have led to the progressive abandonment of the analogic-to-ketones mechanism and formulation of an ‘ionic’ one [32,33,34]. There are other interactions which might take place in the transition state, yet their influence has not been adequately quantified, sufficiently explored in the case of ATH of imines, or is strongly dependent on the structure of the substrate. For instance, several studies suggest that in the disfavored transition state, a destabilizing SO_2_/*π* interaction (repulsion) might take place [50,51] and it is also possible to track down the influence of ‘bulkiness’ of the substrate substituent on the resulting enantiomeric excess [51].

**Figure 5:**
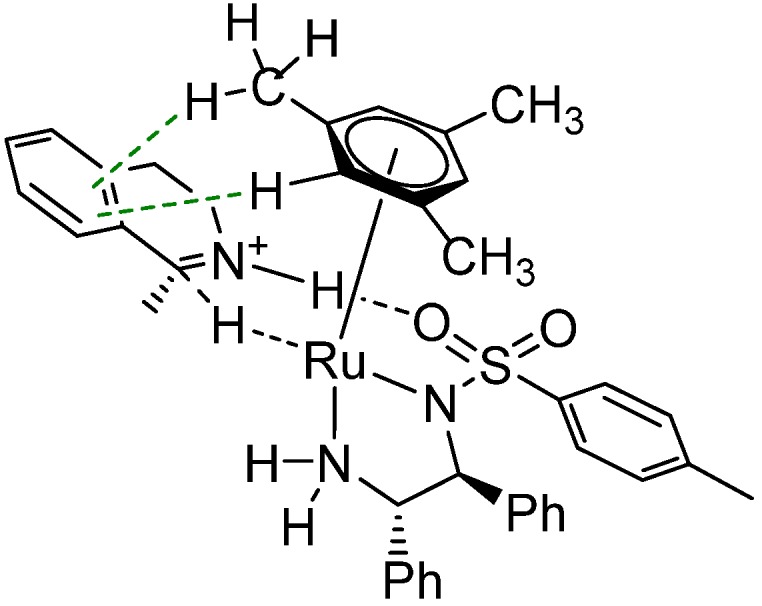
Transition state of hydrogenation of 1-methyl-3,4-DHIQ with [RuH(*η*^6^-mesitylene)TsDPEN] featuring double CH/π interaction.

In the case of ruthenium, only complexes bearing *p*-cymene and mesitylene are commercially available (together with tethered complexes containing the teth-TsDPEN, teth-MsDPEN and teth-TsDENEB ligands) [52]; complexes with benzene or hexamethylbenzene can be prepared from their dimeric precursors of the [RuCl_2_(*η*^6^-arene)]_2_ type, which are commercially available as well. If a different *η*^6^-arene is demanded, then the dimeric precursor needs to be synthesized by a reaction of a corresponding 1,4- or 1,3-cyclohexadiene with (typically hydrated) ruthenium(III) chloride [53]. The dienes can be prepared by Birch reduction of aromatic compounds, or by cycloaddition, as described by the groups of Wills [54] and Ikariya [55]. Analogous dimers with 1,2,3,4,5-pentamethylcyclopentadienyl (Cp*) ligands are available with rhodium and iridium. The half-sandwich complexes can also be synthesized by direct arene exchange, thus avoiding the necessity of having the cyclohexadiene precursor [56,57,58].To the best of our knowledge, for the ATH of imines there is no systematic study on the influence of the structure (*i.e.*, substitution) of the aromatic ligand on both the catalyst itself (geometry, solubility) and the outcome of the hydrogenation reaction (TOF, enantioselectivity). We tested four different catalysts (with benzene, mesitylene, *p*-cymene and hexamethylbenzene) in our recent study [59] and found that catalysts bearing mesitylene and *p*-cymene could deliver higher *ee*s than the catalyst with benzene. Molecular modelling allowed us to investigate the transition states as an extension of our previous work [34]. The structures of the calculated transition states revealed that for mesitylene (Figure 5) and *p*-cymene it was possible to form double CH/π interaction, thus effectively increasing the free energy difference between the *Re* and *Si* paths. Only single CH/π interaction can be formed in the case of benzene, resulting in a lower *ee* value.

Other parameters are affected by the choice of the *η*^6^-arene apart from enantioselectivity [59]. For instance, the turnover frequency (TOF) of the catalyst with hexamethylbenzene was the smallest of all tested catalysts. This might be caused by steric hindrace. The second interesting difference lies in solubility. While catalysts bearing mesitylene, *p*-cymene and hexamethylbenzene were soluble in most polar aprotic solvents, the catalyst with benzene was nearly insoluble. Higher amounts could only be dissolved in dimethyl sulfoxide or *N*,*N*-dimethylformamide, which is sometimes inconvenient owing to relatively high boiling points of these solvents. 

## 3. The Imine Substrate

**Figure 6:**
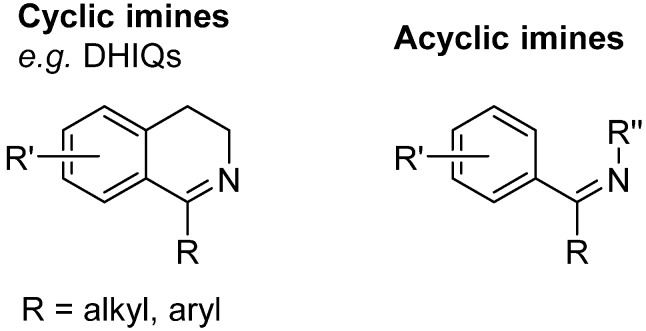
General structures of common imine substrates.

Imines can be divided into several classes according to their structure. According to the position of the C=N bond, cyclic and acyclic imines can be distinguished. The Noyori-Ikariya catalytic system is known to be highly enantioselective especially if the substrates feature an aromatic ring adjacent to the C=N or C=O bond, respectively, in order to facilitate the aforementioned CH/π interaction. On that account, the most common imine substrates for ATH (Figure 6) involve DHIQ derivatives (cyclic imines) and products of dehydration of hemiaminals (acyclic imines). DHIQs usually possess an alkyl or aryl substituent in position 1. While the 1-alkyl-DHIQ derivatives are usually reduced smoothly, 1-aryl-DHIQs are considerably less reactive (see Section 3.1).

### 3.1. 1-Aryl-3,4-Dihydroisoquinolines

1-Aryl substituted THIQs (and DHIQs) have been studied for their activity as e.g., anti-HIV agents [60], inhibitors of c-Jun *N*-terminal kinases [61], anticonvulsant [62] and antispasmodic compounds [63]. However, we consider it somewhat unfortunate that most of these studies evaluated the biological activity of racemic mixtures because we can expect different contributions from both enantiomers. It could be related to the fact that the preparation of chiral 1-aryl substituted THIQs represents a significant challenge. 

An important development in the synthesis of 1-aryl-THIQs was started by Noyori *et al.* in 1996, who performed the ATH of 6,7-dimethoxy-1-phenyl-DHIQ with catalyst [RuCl(*η*^6^-benzene) (NpsDPEN)] (Nps = naphthalene-1-sulfonyl) with great yield and enantioselectivity [13]. Nevertheless, in 1999, Vedejs *et al.* [64] pointed out that substrates lacking methoxy groups are quite non-reactive and hard to hydrogenate with Noyori’s systems. Quite similar results were obtained by Vidal *et al.* in 2013 [18], whose study was focused mainly on 6,7-dimethoxy-1-aryl-DHIQ and its ATH via [RuCl(*η*^6^-benzene)(TsDPEN)]. They also noted that even though [IrClCp*TsDPEN] and [RhClCp*TsDPEN] successfully hydrogenate the aforementioned substrate, the products are racemic. Yu *et al.* [65] tested the hydrogenation of imines with a water-soluble modification of Noyori’s catalyst (based on sulfonylated TsDPEN) and found out that 6,7-dimethoxy-1-aryl-DHIQ did not undergo reduction at all, which was circumvented by reducing an iminium salt (prepared by benzylation of the substrate). It is necessary to point out that a mixture of HCOONa/H_2_O was used as a hydrogen donor. On the other hand, the 1-phenyl-3,4-dihydro-*β*-carboline substrate, which is even more sterically demanding and bears many similarities to 1-aryl-DHIQs, was reduced with high yield and high enantioselectivity.

Naturally, other interesting routes for the hydrogenation of 1-aryl-DHIQs have been developed. A study by Zhang *et al.* shows that [{Ir(COD)Cl}_2_] with f-binaphane as a ligand (and with the addition of iodine/iodic acid) can successfully facilitate the asymmetric hydrogenation (50 bar H_2_) of 1-phenyl-DHIQ with high enantioselectivity [66]. This study was further extended by Núñez-Rico and Vidal-Ferran *et al.* [67] However, phosphine ligands are sensitive to atmospheric oxygen, iridium belongs to the most expensive metals, the pressure of hydrogen is relatively high (although Vidal-Ferran *et al.* showed that 10 bar can be sufficient) and additives are required. Successful ATH of non-methoxylated 1-aryl-DHIQs on Noyori-Ikariya catalysts would bear many advantages from the viewpoints of economics (ruthenium is cheaper than rhodium or iridium), stability (the diamine ligands tend to be quite non-reactive) and safety (avoidance of working with pressurized hydrogen).

Very recently, we set out to investigate the sluggish reactivity of 1-aryl-DHIQs *in silico*. To the best of our knowledge, the non-reactivity of these substrates has not yet been clarified satisfactorily. It is likely related to the bulkiness of the phenyl substituent (*i.e.*, steric effects) and the distribution of electrons through delocalization from the C=N bond, but no evidence has been presented so far.

**Figure 7:**
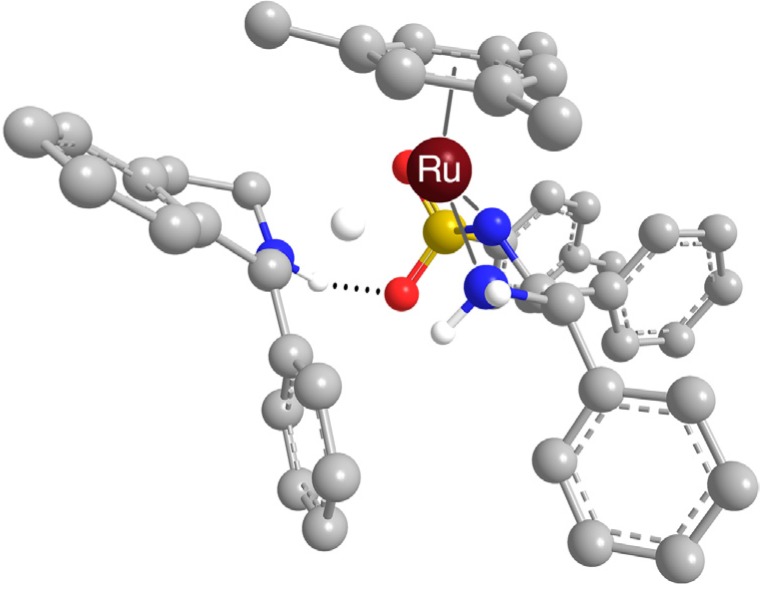
Transition state of hydrogenation of 1-phenyl-DHIQ with [RuH(*η*^6^-mesitylene)(*S*,*S*)-TsDPEN] optimized at the ωB97XD/Def2-SVP level [38].

Figure 7 shows an optimized transition state involving 1-phenyl-3,4-DHIQ and [RuCl(*η*^6^-mesitylene)TsDPEN] (Figure 3, the computational details are given in Supplementary materials, Section 2.3 [38]). Based on our experimental evidence [68], this reaction does not proceed under “standard” conditions used for other substrates [44] (conversion 0.2% after 24 h), but the aforementioned transition state did not show any steric hindrance and all parameters were comparable with our previous studies. This “falsely positive” result suggests that the problem is elsewhere. Previously reported successful hydrogenation of methoxy-containing 1-aryl-DHIQs on [RuCl(*η*^6^-benzene)NpsDPEN] and 1-aryl-*β*-3,4-dihydrocarbolines on [RuCl(*η*^6^-*p*-cymene)TsDPEN] also supports our hypothesis that the non-reactivity is not caused by steric effects (either related to the substrate, or to the catalyst).

It has been confirmed several times that the imine substrate must be protonated in order to be able to undergo reduction, so we decided to investigate the interaction between the imine molecule and formic acid as a potential source of non-reactivity. Because we expected the formation of anions, we included diffuse functions (by using the Def2-SVPD basis set [69]), yet this complicated the convergence of the geometry and was found significantly more hardware-demanding. The IEFPCM solvation model (acetonitrile) was used for all calculations, including geometry optimizations.

Our results (Table 3, Section 2.2 in Supplementary materials [38]) indicate that the substitution of the DHIQ backbone leads to striking differences in interaction with formic acid. Substrates containing a methoxy group in position 6 or 7 (or both) were able to attract the acidic hydrogen towards the imine nitrogen and form cationic and anionic species. On the other hand, the substrate with a bulky phenyl substituent in position 1 was unable to attract the acidic hydrogen and acceptor/donor relationship based on a hydrogen bond was observed instead. 

**Table 3:** Calculated interatomic distances for the proton located between the nitrogen of the imine substrate and oxygen of the formate anion [38] ^[a]^. 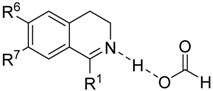

	1-Me	6-MeO-1-Me	7-MeO-1-Me	6,7-diMeO-1-Me	1-Ph
N–H [Å]	1.5196	1.0675	1.0763	1.0702	1.5677
H–O [Å]	1.0522	1.5868	1.5556	1.5764	1.0416
C=N [Å] ^[a]^	1.2785	1.2799	1.2784	1.2788	1.2795
C=N [Å] ^[b]^	1.2825	1.2943	1.2900	1.2929	1.2828
C=N^+^ [Å] ^[c]^	1.2977	1.3026	1.2978	1.3020	1.3020
Activation [%] ^[d]^	21.3	63.4	59.8	60.8	14.7

^[a]^ DHIQ without formic acid. ^[b]^ DHIQ forming an associate with formic acid. ^[c]^ Fully protonated DHIQ (anion omitted). ^[d]^ Activation was calculated by comparing the C=N bond length difference (between neutral DHIQ and DHIQ interacting with formate) with the difference between neutral DHIQ and theoretical maximal activation represented by protonated DHIQ (without the counter-anion).

The results for 1-phenyl-3,4-DHIQ are quite similar to 1-methyl-3,4-DHIQ, whose interaction with formic acid also appears to be based on hydrogen bonding, which is quite unexpected, because 1-methyl-DHIQ can be successfully reduced, while the reduction of the other substrate is quite problematic (as described above). Further evidence is being gathered. One of the possible explanations is the level of ‘degeneration’ caused by protonation. In the case of 1-methyl-DHIQ, the C=N double bond elongates upon protonation up to 21% of the theoretical maximum (based on the geometry of an isolated iminium cation), and for 1-Ph-DHIQ it degenerates only up to 14%. On the other hand, the C=N bond within methoxyl-containing substrates elongates up to 60% of the maximum, which suggests extremely strong activation. The results thus indicate that 1-phenyl-3,4-DHIQ, which is quite hard to hydrogenate, might be able to undergo successful hydrogenation in the case of its successful activation, which is probably not achievable by formic acid.

3.2. 1-Benzyl-3,4-dihydroisoquinolines

1-Benzyl-DHIQ derivatives, which can be ranked among 1-alkyl-DHIQs, represent an interesting group of compounds. The ATH of such substrates is typical for the production of muscle relaxants such as mivacurium chloride or gantacurium chloride [70,71]. Their reactivity is comparable with that of 1-alkyl-DHIQs (*i.e.*, higher than the reactivity of 1-Ph-DHIQs), suggesting that the steric effects of the substituents (benzyl *vs.* phenyl) in position 1 are not the key parameter regarding the ATH reaction rate.

**Scheme 2:**
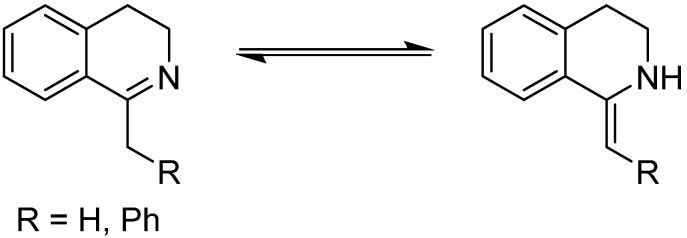
Tautomerization of DHIQs.

DHIQs can undergo imine-enamine tautomerization (Scheme 2). We have observed a consequence of this phenomenon in ^1^H NMR spectra measured in methanol-*d*_4_, where the signal of the CH_2_ group was missing due to the prompt H/D exchange [72]. This contrasts with a 1-methyl-3,4-DHIQ derivative (*i.e.*, a compound missing the additional phenyl ring adjacent to the C=N bond), which exhibited this behaviour only in the presence of *p*-toluenesulfonic acid [73]. However, as noted by Noyori *et al.* in their original paper [13], the hydrogenation of simple olefins does not proceed with this catalytic system, and therefore only the ketimine form of the substrate undergoes reduction.

An interesting extension of this chemistry is the oxidation of the methylene group to a ketone as reported by Martin *et al.* [74]. The oxidation occurred even with air oxygen in benzene, but the best result was obtained with pure O_2_ in propan-2-ol using methylene blue and UV/Vis irradiation to generate singlet oxygen (^1^O_2_). The oxidation presumably takes places via a charge-transfer mechanism [75]. The authors also report the cleavage of the H_2_*C*–*C*=N bond in the case of substrates bearing substituents in the benzylic position or at the nitrogen atom, resulting in two carbonyl products [76], which has been shown for enamines in general [77]. This special behaviour of 1-benzyl-DHIQs should therefore be taken into account when operating in non-degassed solvents or in the presence of oxidizing reagents.

Along with other examples of air oxidation [78,79,80,81] and oxidation with singlet oxygen as described above, many modified or different approaches have been reported, using inorganic oxidizing agents (MnO_2_ [82], SeO_2_ [83]), palladium on charcoal [84,85], platinum on charcoal [86], or even metabolism of *Pseudomonas putida* [87]. The introduction of the C=O functionality extends the possibilities of structural modifications of DHIQ substrates by all reactions based on the reactivity of a ketone group.

#### 3.3. Acyclic Imines

**Scheme 3:**
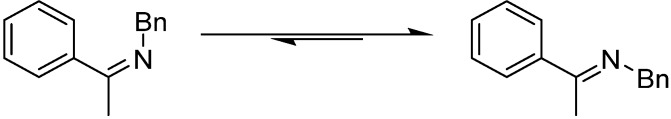
Isomerization of acetophenone *N*-benzylimine.

The ATH of acyclic imines represents a great challenge for the examined class of catalysts. This is mainly due to the significant difference in their backbone – while the C=N bond of cyclic substrates like DHIQs and *β*-carbolines is fixed within a rigid structure, acyclic substrates like acetophenone *N*-benzylimine have an exocyclic C=N bond, which therefore permits an *E*/*Z* isomerisation (Scheme 3). Each geometrical isomer can thus interact with the catalyst differently. Theoretically, there are only two possibilities: 1) both isomers are reduced, yet the structural diversity leads to the fact that they can be reduced with different enantioselectivity, or 2) only one of the two isomers is reduced (*i.e.*, due to steric hindrance). Fortunately, the energy barrier of the *E*/*Z* conversion is sufficiently low to permit the transformation, thus effectively solving the problems associated with the second option. The isomerisation can also be catalyzed by acidic conditions, but on the other hand, in our experience based on working with acetophenone *N*-benzylimine, the compound is prone to decomposition under acidic conditions (but this is also dependent on concentration and time).

Despite all the aforementioned difficulties, Noyori *et al.* demonstrated on the example of several acyclic substrates that they can be successfully reduced while maintaining relatively high enantioselectivity, but lower than in the case of cyclic substrates [13]. Another intriguing observation of theirs is that the majority of the resulting amines (after hydrogenation with a catalyst with the (*S,S*)-ligand) bear the (*S*)-configuration on the asymmetric carbon. This seemingly appears to defy the contemporary conception of the mechanism formulated on experiments with DHIQs (*i.e.*, cyclic derivatives), the reduction of which proceeds at the opposite face [33,34]. We investigated this phenomenon as a part of our previous computational study on acetophenone *N*-benzylimine [88]. It appears that it is simply a “native” feature of this type of backbone –*i.e.*, the substrate attains a slightly different conformation (which allows it to minimize its energy) but because of that, the opposite isomer of the resulting amine is formed; apart from that, all other features closely follow the currently proposed ionic mechanism.

Several studies have addressed the possibility of synthesis of substances which are interesting from the industrial point of view [10]. For instance, Noyori *et al.* showed that it is viable to employ their catalyst system for the preparation of an intermediate of sezolamide (previously MK-417), a carbonic anhydrase inhibitor. Fan *et al.* demonstrated a possible synthetic pathway for the preparation of (+)-sertraline, a chiral antidepressant drug [89]. Nevertheless, their ‘standard’ procedure was heavily modified – among the most prominent changes belong the usage of gaseous hydrogen instead of a liquid donor, employment of catalysts bearing various weakly-coordinating anions (especially BAr^F^) instead of chloride [90], and usage of di-*tert*-butyl dicarbonate (Boc_2_O) as an additive preventing benzylamine (a minor by-product) from inhibiting the catalyst.

## 4. Conclusions

The purpose of this review was to present a compilation of selected topics on the Noyori-Ikariya enantioselective reduction of imines with the [RuCl(*η*^6^-arene)(*N*-arylsulfonyl-DPEN)] catalysts. Our attention was particularly aimed at the importance of the sulfonyl and *η*^6^-arene moieties of the catalysts, which play an important role in the reaction mechanism. In the next section, the structural features of common imine substrates were described and discussed. Nearly all sub-chapters comprise both experimental and theoretical findings – in this way, we wish to show the symbiotic approach of these two contrasting methodologies, which is sometimes closer and sometimes further from the ideal case of a perfect match. The work is a deliberate follow-up of our previous two *Molecules* reviews [9,10], which together try to attract attention to the new ideas and concepts that have been recently reported, and to show that this topic is still “hot”.

## Supplementary Materials

Supplementary materials can be accessed at: http://www.mdpi.com/1420-3049/19/6/6987/s1.
