# Supplementary material for: Experimental and Theoretical Perspectives of the Noyori-Ikariya Asymmetric Transfer Hydrogenation of Imines
Source: Molecules. 2014 May 28;19(6):6987–7007. doi: 10.3390/molecules19066987 (PMC6272002; doi:10.3390/molecules19066987)
Supplement: Supplementary file 1 [file molecules-19-06987-s001.pdf]

Supplementary Materials

1. Experimental Data

All commercially available chemicals were used without further purification. The following compounds were prepared according to literature: 1-methyl-3,4-DHIQ [1], 6-methoxy-1-methyl-3,4-DHIQ, 7-methoxy-1-methyl-3,4-DHIQ, 6,7-dimethoxy-1-methyl-3,4-DHIQ [2], 1-phenyl-3,4-DHIQ [3].

The kinetic experiments (Table 2) were performed in a round−bottom flask as reported previously [1]. The *ee* values were determined by a pre−column GC derivatization method [4].

Synthesis of Piperidine-^{15}N:

Pyridine-^{15}N (255 µL, 3.2 mmol) was dissolved in MeOH (2 mL) and Ru/C catalyst (20% *w/w*, supplied by Johnson Matthey, type 97) was added thereto. The reduction was done in a Parr pressure batch reactor with a glass inlet (60 °C, 4.5 h, $p(H_2)$ = 70 bar). After cooling to RT, HCl in MeOH was added to the mixture and MeOH was evaporated. The solution of the hydrochloride salt was alkalized with aqueous NaOH and the product was extracted to diethyl ether.

Preparation of the NMR Sample for gHMBC Spectra Measurement:

Catalyst [RuCl(η^6-*p*-cymene)TsDPEN] (7 mg, 0.011 mmol) was dissolved in acetonitrile-d_3 (0.5 mL). Piperidine-^{15}N (10.9 µL, 0.110 mmol) was added thereto, followed by HCOOH (10.4 µL, 0.275 mmol).

2. Computational Data

This section lists the geometries of all calculated species and detailed description of the computational methods used in this work.

2.1. Calculation of Catalyst−Amine Associates (Table 1):

The geometries were optimized on the density functional theory (DFT) level employing the *Gaussian 09* suite [5]. The ωB97XD [6] functional was used with the Def2-SVP basis set for all atoms [7] and effective core potential for Ru [8]. The calculations of single−point energies were done both on the DFT and MP2 level (the DFT//DFT and MP2//DFT approach, respectively). The IEFPCM solvation model was applied using acetonitrile with default parameters.

**NAME:**	unsolvated [Ru]-H
**SINGLE−POINT ENERGY (DFT):**	−1954.42378213 hartree
**CHARGE, MULTIPLICITY:**	0, 1
**STRUCTURE TYPE:**	MINIMUM
**COMMENT:**	…

C	−0.51152300	0.98996800	−0.61339300
H	−0.61898900	1.40630700	−1.63412500
C	0.26777200	2.02500400	0.21613200
H	0.33329100	1.64146400	1.24599000

N	1.64905600	2.05599900	−0.29731000
H	1.66139300	2.40769400	−1.25435000
H	2.21276600	2.70133800	0.25493200
N	0.27376900	−0.24393100	−0.64323700
C	−0.39870800	3.38313900	0.24012100
C	−1.02067400	3.84071900	1.40401200
C	−0.45098700	4.17906100	−0.90982900
C	−1.67943600	5.06868900	1.42382400
H	−1.00587400	3.21558600	2.30061800
C	−1.10367100	5.40941700	−0.89266300
H	0.00497200	3.83245100	−1.84197100
C	−1.71998700	5.85732600	0.27558700
H	−2.16630300	5.40910800	2.34004000
H	−1.13792700	6.01799000	−1.79893500
H	−2.23544900	6.81994300	0.28854900
C	−1.90850800	0.84955400	−0.02419200
C	−2.98974800	1.52352400	−0.59375600
C	−2.12145100	0.07907900	1.12118500
C	−4.26266500	1.42672700	−0.03362900
H	−2.83281500	2.12492000	−1.49287300
C	−3.39111500	−0.02625200	1.68065800
H	−1.28142000	−0.46921000	1.54933100
C	−4.46768800	0.64900200	1.10419100
H	−5.10034600	1.95526000	−0.49423000
H	−3.54435000	−0.64731700	2.56621800
H	−5.46568100	0.56420100	1.54045600
S	−0.17257000	−1.26089200	−1.85656300
O	−0.56791400	−0.51930600	−3.05698400
O	0.82153800	−2.32417100	−1.97749300
C	−1.65681700	−2.05456500	−1.24281400
C	−1.55292700	−3.01630400	−0.24211500
C	−2.90275200	−1.66340000	−1.72089400
C	−2.70973400	−3.55217700	0.31541400
H	−0.56790500	−3.34267600	0.09697300
C	−4.05231500	−2.20489600	−1.15545600
H	−2.95590100	−0.92156700	−2.51853500
C	−3.97607600	−3.14258700	−0.11917400
H	−2.62867900	−4.30288000	1.10609400
H	−5.03129000	−1.88063200	−1.51812300
C	−5.23222100	−3.67365900	0.51864000
H	−5.94323900	−4.03783200	−0.23815500
H	−5.01834400	−4.49894900	1.21199800
H	−5.74069900	−2.87840500	1.08761200
Ru	2.37185500	0.03980800	−0.34059300
H	5.34114100	−0.18770200	−2.26593300
C	5.59215600	0.03068900	−1.21941600
C	4.49152400	−0.42284900	−0.30386200
H	5.76286800	1.11213400	−1.12588600
H	6.53368700	−0.48676900	−0.97570500
C	4.15106400	0.29513000	0.86983800
C	3.73076200	−1.60654200	−0.58308900

C	3.16094000	−0.17122500	1.80351200
H	4.67137300	1.23367100	1.07882500
C	2.69164600	−1.99993800	0.28319600
H	3.88011800	−2.15241700	−1.51498400
C	2.40866200	−1.31386200	1.51838500
H	2.94972200	0.42802800	2.69008900
H	2.04697100	−2.81833800	−0.03631600
C	1.22818400	−1.78663500	2.33924800
H	0.40722200	−1.87565300	1.60632000
C	0.78080800	−0.79745800	3.41035500
C	1.49009300	−3.17250100	2.93735500
H	0.56880000	0.19313200	2.98073600
H	−0.14073900	−1.15329400	3.89412200
H	1.54210300	−0.67585500	4.19780000
H	0.58810400	−3.55413500	3.43939200
H	1.77724500	−3.90028600	2.16429600
H	2.30253000	−3.13296600	3.68032500
H	2.43471800	0.32663100	−1.90407500

**NAME:**	unsolvated butylamine-H+
**SINGLE−POINT ENERGY (DFT):**	−213.95502144 hartree
**CHARGE, MULTIPLICITY:**	1, 1
**STRUCTURE TYPE:**	MINIMUM
**COMMENT:**	…

C	−2.58379500	−0.32633800	−0.00001000
H	−2.63184700	−0.97146400	−0.89030500
H	−2.63181900	−0.97157200	0.89020700
H	−3.47986600	0.30863500	0.00003700
C	−1.32050600	0.52646600	0.00001400
H	−1.31572700	1.18759900	−0.88261300
H	−1.31573600	1.18756600	0.88266700
C	−0.04524700	−0.32097400	0.00001100
H	−0.04235300	−0.97814400	0.88773000
H	−0.04236800	−0.97816400	−0.88769300
C	1.20196500	0.54403500	−0.00001300
H	1.26085800	1.18472100	−0.89079200
H	1.26086800	1.18476100	0.89073700
N	2.44987300	−0.30339600	0.00000000
H	2.48336900	−0.91598400	−0.82543600
H	3.30764100	0.26263500	−0.00001100
H	2.48337200	−0.91595400	0.82545900

**NAME:**	unsolvated piperidine-H+
**SINGLE−POINT ENERGY (DFT):**	−252.03681413 hartree
**CHARGE, MULTIPLICITY:**	1, 1
**STRUCTURE TYPE:**	MINIMUM
**COMMENT:**	…

C	−0.74713700	1.26029500	−0.22565900
C	−1.48795800	0.00013200	0.22386400
C	−0.74736800	−1.26016500	−0.22565500
C	0.69990800	−1.25747200	0.23989600
C	0.70013300	1.25735000	0.23990000
H	−1.22652000	−2.16583200	0.17214900
H	−1.58762000	0.00014200	1.32218600
H	−2.50851600	0.00022900	−0.18191200
H	−0.77970200	1.35438400	−1.32504100
H	−1.22614500	2.16604500	0.17213100
H	0.77573900	−1.26033900	1.33702800
H	1.27367900	−2.10793600	−0.15154900
H	2.36493300	−0.00020900	0.09093200
H	1.42367000	−0.00012100	−1.25052200
H	0.77595500	1.26019900	1.33703300
H	1.27406100	2.10771200	−0.15153800
H	−0.77995400	−1.35426000	−1.32503600
N	1.38785100	−0.00012200	−0.22284800

**NAME:**	unsolvated triethylamine-H+
**SINGLE−POINT ENERGY (DFT):**	−292.50960268 hartree
**CHARGE, MULTIPLICITY:**	1, 1
**STRUCTURE TYPE:**	MINIMUM
**COMMENT:**	…

N	0.05479400	−0.20665600	0.65868800
C	−1.40904500	−0.60508100	0.69020400
H	−1.44552400	−1.55615400	1.24082900
H	−1.91369300	0.15196100	1.30582800
C	−2.08632600	−0.76454600	−0.65773700
H	−3.14727500	−0.97909100	−0.46916800
H	−2.04031200	0.14024200	−1.27547000
H	−1.68474800	−1.60959400	−1.23181900
C	0.29761600	1.28545300	0.65496400
H	1.36390400	1.41729600	0.88103300
H	−0.26549900	1.67931600	1.51246500
C	−0.07470200	2.01034100	−0.62106700
H	−1.15675300	2.01897800	−0.80240600
H	0.24251900	3.05667000	−0.51407700

H	0.43744900	1.60602900	−1.50515800
C	0.84814300	−0.97466200	−0.36402700
H	0.57116300	−2.02924700	−0.22370100
H	0.48999800	−0.66701300	−1.35390700
C	2.34502100	−0.78523900	−0.22426300
H	2.69764600	−1.01219400	0.79385900
H	2.84807600	−1.48447600	−0.90543600
H	2.67248900	0.22672600	−0.49825600
H	0.42276300	−0.51044500	1.56612800

**NAME:**	unsolvated tributylamine-H+
**SINGLE−POINT ENERGY (DFT):**	−528.16659873 hartree
**CHARGE, MULTIPLICITY:**	1, 1
**STRUCTURE TYPE:**	MINIMUM
**COMMENT:**	…

C	4.40549700	0.62280800	−0.41533700
H	3.79481900	1.45037400	−0.80902700
H	4.68046900	0.86606700	0.62310800
H	5.33227000	0.60645200	−1.00501700
C	3.69370300	−0.72435900	−0.49837100
H	3.39651500	−0.93231100	−1.54004000
H	4.39584900	−1.52494900	−0.22217800
C	2.46738400	−0.85580900	0.41139600
H	2.08679700	−1.88716200	0.34947100
H	2.77337800	−0.68944000	1.46052100
C	1.36616400	0.12821700	0.04925000
H	1.72258400	1.16367500	0.13735100
H	1.01184900	−0.01613900	−0.97878500
N	0.18016600	0.01982000	0.96585100
H	0.57830100	0.03077300	1.91017700
C	−0.71146100	1.23625300	0.93014900
H	−1.40136300	1.13592400	1.78123300
H	−0.05097900	2.09000700	1.13737800
C	−1.48671800	1.44748800	−0.35904400
H	−0.80114500	1.44131200	−1.22245500
H	−2.20045000	0.62224300	−0.50055400
C	−2.26169200	2.77147500	−0.34319200
H	−2.89545600	2.78903500	−1.24242600
H	−2.95820400	2.77721100	0.51210900
C	−1.38844700	4.02259800	−0.31754100
H	−2.00521800	4.92545500	−0.42366900
H	−0.83052500	4.13283600	0.62553000
H	−0.66178600	4.02081900	−1.14510900
C	−0.56849000	−1.29483300	0.88338700
H	−0.01277400	−2.00958300	1.50673700
H	−1.53259800	−1.11448900	1.37749400
C	−0.76090300	−1.87241800	−0.51221600

H	−1.14032200	−1.10894900	−1.20726900
H	0.20779300	−2.21070200	−0.90988400
C	−1.73226800	−3.05959300	−0.49126200
H	−1.66454300	−3.55866800	−1.46917700
H	−1.38790700	−3.80324100	0.24721500
C	−3.18564000	−2.68229200	−0.21976600
H	−3.55320600	−1.95564300	−0.96134700
H	−3.83317400	−3.56787200	−0.27893200
H	−3.33491200	−2.24898800	0.78186100

**NAME:**	unsolvated [Ru]-H + butylamine-H+
**SINGLE−POINT ENERGY (DFT):**	−2168.46813953 hartree
**CHARGE, MULTIPLICITY:**	1, 1
**STRUCTURE TYPE:**	MINIMUM
**COMMENT:**	…

C	0.80204800	1.07508400	−0.71072300
H	0.63298900	1.16496600	−1.80079700
C	2.32250200	1.14073300	−0.48671700
H	2.49611300	1.11078300	0.59947600
N	2.91123200	−0.09828300	−1.03218900
H	2.80478300	−0.12159500	−2.04632300
H	3.91613800	−0.10539800	−0.85633100
N	0.34137200	−0.24070200	−0.23530900
C	2.93799300	2.40508200	−1.04358300
C	3.37099600	3.41001000	−0.17545900
C	3.03705700	2.61321600	−2.42383200
C	3.89593700	4.60070600	−0.67385600
H	3.27925000	3.26540400	0.90402200
C	3.56715900	3.79984400	−2.92525100
H	2.68815400	1.85143100	−3.12810200
C	3.99740400	4.79672300	−2.04972800
H	4.22679700	5.37854700	0.01697300
H	3.64273900	3.94929400	−4.00413400
H	4.41177600	5.72737800	−2.44237300
C	0.14149200	2.26536300	−0.03462600
C	−0.19033400	3.40045200	−0.77589500
C	−0.10190800	2.25792600	1.34080900
C	−0.76589000	4.50839100	−0.15597100
H	0.00520000	3.41934100	−1.85130400
C	−0.68415500	3.35861800	1.96235500
H	0.14038100	1.36388600	1.91752500
C	−1.01876500	4.48810500	1.21429000
H	−1.01957500	5.39098800	−0.74688200
H	−0.88094500	3.33450400	3.03638400
H	−1.47356300	5.35368800	1.70064800
S	−1.11646100	−0.64920000	−0.74697500
O	−1.34781800	−0.34023700	−2.18749100

O	−1.39004900	−2.06605000	−0.40007500
C	−2.31687000	0.30480100	0.15384400
C	−2.61482200	−0.06347300	1.46304100
C	−2.90590100	1.42363200	−0.42856200
C	−3.50159500	0.71413800	2.20075200
H	−2.15870700	−0.95312000	1.90147800
C	−3.78456000	2.19330000	0.32370400
H	−2.66280600	1.69221200	−1.45716000
C	−4.09019300	1.85928200	1.64969200
H	−3.73645900	0.43002100	3.22923200
H	−4.23565500	3.08122000	−0.12548900
C	−5.01370000	2.72822700	2.45786300
H	−5.97059900	2.88342300	1.93779100
H	−5.22601500	2.29049400	3.44222500
H	−4.56350700	3.72060300	2.61675200
Ru	1.86987900	−1.76295100	−0.17742300
H	2.60424500	−4.84774100	−1.83532300
C	3.35228200	−4.63204900	−1.06122500
C	2.78601500	−3.72772800	−0.00538700
H	4.22748800	−4.17814100	−1.54500600
H	3.66768100	−5.58625500	−0.61147900
C	3.58720200	−2.77186900	0.66571000
C	1.40545500	−3.79447800	0.37521900
C	3.09065300	−1.96982800	1.75011900
H	4.62952000	−2.65515800	0.35868700
C	0.90155800	−2.92718500	1.36400000
H	0.72342600	−4.46220500	−0.15152100
C	1.74205200	−2.02728800	2.11002500
H	3.76547500	−1.26125100	2.23146500
H	−0.17386200	−2.92072900	1.54342100
C	1.09806500	−1.14449800	3.15777300
H	0.19903400	−0.72834200	2.66910000
C	1.96821900	0.02619400	3.60415000
C	0.64461100	−1.98623700	4.35678700
H	2.30477200	0.63341100	2.75065400
H	1.39984400	0.68537500	4.27586700
H	2.85770700	−0.31524100	4.15620600
H	0.10882200	−1.36168700	5.08669500
H	−0.02823700	−2.80133700	4.05208100
H	1.51037800	−2.43733800	4.86572800
H	1.48541400	−2.05253800	−1.70184600
C	−6.39856900	−0.56139500	−0.63666100
H	−5.92913200	−0.70684200	0.34914900
H	−6.10588600	0.43827600	−0.99309300
H	−7.48876200	−0.56631200	−0.49865800
C	−5.95851100	−1.64282400	−1.61810100
H	−6.31441300	−2.62838700	−1.27262200
H	−6.43080000	−1.47177500	−2.60039300
C	−4.44205300	−1.67386800	−1.77190700
H	−4.07975800	−0.68208700	−2.08797500
H	−3.97716000	−1.86563400	−0.79146800

C	−3.94911400	−2.71538000	−2.76324800
H	−4.27461900	−3.72757000	−2.48258700
H	−4.31442700	−2.51178700	−3.78004300
N	−2.46093400	−2.71596600	−2.80607500
H	−2.05551900	−2.88063900	−1.85433800
H	−2.07079900	−3.37966200	−3.47741300
H	−2.07753000	−1.75485000	−2.98155900

**NAME:**	unsolvated [Ru]-H + piperidine-H+ (1)
**SINGLE−POINT ENERGY (DFT):**	−2206.54515885 hartree
**CHARGE, MULTIPLICITY:**	1, 1
**STRUCTURE TYPE:**	MINIMUM
**COMMENT:**	…

C	0.96532600	−0.44746100	−0.84470100
H	0.62494900	−0.12684400	−1.84883400
C	0.96235900	−1.98498300	−0.83769200
H	1.33882400	−2.30768500	0.14420100
N	−0.44341600	−2.43422600	−0.90683600
H	−0.82961300	−2.21928900	−1.82578200
H	−0.47918400	−3.45068900	−0.82405300
N	0.01108200	0.00110100	0.17978000
C	1.83665900	−2.58201500	−1.91723400
C	3.02241500	−3.23523600	−1.57458600
C	1.50582500	−2.44475100	−3.27024900
C	3.86471100	−3.74199600	−2.56236100
H	3.29931700	−3.33226100	−0.52172200
C	2.34277700	−2.95427500	−4.25985600
H	0.59368400	−1.91801700	−3.56850000
C	3.52609000	−3.60296300	−3.90676600
H	4.79177500	−4.24463300	−2.28025900
H	2.07605300	−2.83766700	−5.31216800
H	4.18626000	−3.99729700	−4.68171500
C	2.39406800	0.03822100	−0.65898700
C	3.15991300	0.40396800	−1.76705400
C	2.97804800	0.06708700	0.60920500
C	4.48735900	0.79875500	−1.61236800
H	2.71267900	0.37713600	−2.76403700
C	4.30137200	0.46804400	0.76892800
H	2.37450700	−0.19897600	1.47892300
C	5.06040000	0.83526000	−0.34284600
H	5.07531200	1.08172300	−2.48799200
H	4.74319200	0.49999200	1.76719800
H	6.09918000	1.14819100	−0.21820900
S	−0.41835200	1.55504000	0.05595700
O	−0.71217100	1.93251400	−1.36075100
O	−1.52352100	1.82428700	0.98476300
C	0.95595600	2.55052100	0.56150300

C	1.21855900	2.66480100	1.92582400
C	1.78380700	3.14672500	−0.38325700
C	2.34591300	3.36252000	2.33976500
H	0.54075800	2.21742000	2.65535900
C	2.91268300	3.83534300	0.04840600
H	1.55287600	3.05484000	−1.44476000
C	3.22038800	3.94238900	1.40947400
H	2.55422100	3.46024800	3.40789100
H	3.57449200	4.29130200	−0.69133100
C	4.47745100	4.63232700	1.86188400
H	4.78983300	5.41012300	1.15181700
H	4.35307000	5.09474300	2.85087500
H	5.30094700	3.90350300	1.93671400
Ru	−1.56243100	−1.41201400	0.59814900
H	−5.13044300	−1.64604300	0.21949000
C	−4.71979600	−2.53176000	0.72296000
C	−3.42332100	−2.20924700	1.40767500
H	−4.58870800	−3.32389600	−0.02625400
H	−5.46154000	−2.87671600	1.45967200
C	−2.39654100	−3.17287000	1.54666100
C	−3.17261600	−0.90785900	1.95557500
C	−1.18314300	−2.90047200	2.26570300
H	−2.53487700	−4.15739100	1.09340400
C	−1.94547000	−0.62616000	2.58471100
H	−3.90869000	−0.11038300	1.84295200
C	−0.93561400	−1.62783800	2.79207700
H	−0.42406200	−3.68088900	2.33093200
H	−1.74470000	0.40273000	2.88200000
C	0.35566900	−1.21690900	3.46703900
H	0.66166800	−0.28961400	2.95080300
C	1.48489400	−2.23070000	3.31429900
C	0.10265900	−0.88713900	4.94309100
H	1.68110900	−2.46740300	2.25807700
H	2.41476400	−1.82655100	3.73921800
H	1.26483600	−3.17050200	3.84455000
H	1.02099100	−0.50885000	5.41534600
H	−0.67706700	−0.12084900	5.06402600
H	−0.21790900	−1.78445200	5.49476000
H	−2.33663900	−0.82596100	−0.68962400
C	−3.85404000	3.87979800	−1.82279700
C	−4.49020900	3.45529200	−3.14785700
C	−3.89867100	2.13229100	−3.63924000
C	−4.00513200	1.05205400	−2.57186900
C	−3.96751100	2.78103100	−0.77635800
H	−4.40825100	1.78379900	−4.54886500
H	−5.58001600	3.34272500	−3.01405500
H	−4.34742200	4.23728600	−3.90702300
H	−2.78583000	4.11071100	−1.97166000
H	−4.32537000	4.79195700	−1.43054100
H	−5.05661800	0.81209900	−2.34946500
H	−3.49404600	0.12373800	−2.86128100

H	−3.34383500	0.77827800	−0.61161000
H	−2.34534300	1.70949200	−1.48822700
H	−5.01830300	2.57129800	−0.52251000
H	−3.41348900	3.01518800	0.14125000
H	−2.83626200	2.27119700	−3.90151700
N	−3.37486100	1.52968600	−1.31803700

**NAME:**	unsolvated [Ru]-H + piperidine-H+ (2)
**SINGLE−POINT ENERGY (DFT):**	−2206.54084443 hartree
**CHARGE, MULTIPLICITY:**	1, 1
**STRUCTURE TYPE:**	MINIMUM
**COMMENT:**	…

C	0.72001300	1.04337800	−0.68602200
H	0.36602000	1.07509300	−1.73438100
C	2.25773300	1.09711600	−0.72915100
H	2.61356700	1.12921700	0.31189900
N	2.74122400	−0.17914300	−1.28974500
H	2.47446800	−0.25535200	−2.27112100
H	3.76122200	−0.19222900	−1.27613000
N	0.33672000	−0.23300000	−0.06276300
C	2.77866700	2.31318300	−1.46291100
C	3.39512700	3.34784500	−0.75594600
C	2.61119500	2.44484400	−2.84623300
C	3.83735900	4.49300700	−1.41560300
H	3.51662600	3.26243500	0.32686800
C	3.05641400	3.58571700	−3.50939600
H	2.11493300	1.65745700	−3.42241600
C	3.66999200	4.61377600	−2.79361900
H	4.31406300	5.29582800	−0.84980300
H	2.91965600	3.67661600	−4.58882500
H	4.01745800	5.50990800	−3.31139700
C	0.20475600	2.28642600	0.02205600
C	−0.20666300	3.39700300	−0.71694300
C	0.18443600	2.35461500	1.41687900
C	−0.64187200	4.55476600	−0.07463900
H	−0.18268000	3.35680300	−1.80918400
C	−0.25652400	3.50632200	2.06253500
H	0.48931200	1.48082200	1.99499400
C	−0.67227300	4.61059500	1.31754100
H	−0.95899500	5.41708300	−0.66489500
H	−0.28024500	3.54202700	3.15390000
H	−1.01651300	5.51556000	1.82266300
S	−1.18961300	−0.66041100	−0.29359900
O	−1.63719600	−0.42054700	−1.70651600
O	−1.43374600	−2.02717500	0.17732000
C	−2.22179100	0.40064500	0.69596900
C	−2.34931100	0.12183700	2.05444000

C	−2.84704500	1.51006700	0.13213600
C	−3.09209500	0.98174900	2.85697700
H	−1.87448800	−0.76495500	2.47853100
C	−3.58279700	2.36138800	0.94744800
H	−2.73033400	1.71528200	−0.93244200
C	−3.70815300	2.12006200	2.32194600
H	−3.19449100	0.76637900	3.92317200
H	−4.05721500	3.24294400	0.50980900
C	−4.46987500	3.07896000	3.19474100
H	−5.44834400	3.32483200	2.75707200
H	−4.63537500	2.67029600	4.20047300
H	−3.91347500	4.02375500	3.30074700
Ru	1.83671700	−1.77977200	−0.19665900
H	2.38096300	−4.90396400	−1.86182700
C	3.22918100	−4.64588300	−1.21415900
C	2.79940500	−3.72610900	−0.10833800
H	4.00721000	−4.18170800	−1.83512000
H	3.63849700	−5.57818400	−0.79491700
C	3.66920100	−2.74150600	0.42234200
C	1.48789100	−3.80421800	0.46574500
C	3.31625200	−1.91887600	1.54611900
H	4.65475200	−2.61642300	−0.03352700
C	1.11002400	−2.92337700	1.49664900
H	0.74929100	−4.49250200	0.05373000
C	2.02889700	−1.98373000	2.08594900
H	4.03843700	−1.18957400	1.91397000
H	0.07087000	−2.92810700	1.82463300
C	1.51206800	−1.05952600	3.16855700
H	0.57492600	−0.64202300	2.75850900
C	2.44190100	0.10857100	3.48111600
C	1.17048500	−1.85082900	4.43655600
H	2.69086400	0.68442900	2.57710900
H	1.96098100	0.79746500	4.19052200
H	3.38184800	−0.23264900	3.94250900
H	0.72510900	−1.18993500	5.19484900
H	0.45449500	−2.66065200	4.23341300
H	2.07549100	−2.30300900	4.87091300
H	1.19917900	−2.12327800	−1.62046100
C	−4.74873700	−2.08656000	−0.64110200
C	−5.73486900	−1.27563100	−1.47997100
C	−5.01019000	−0.55897600	−2.61858200
C	−4.21880000	−1.53892300	−3.47220900
C	−3.92113700	−3.03699800	−1.49317200
H	−5.71711800	−0.02337200	−3.26804300
H	−6.51045000	−1.94207900	−1.89572900
H	−6.25511300	−0.54478100	−0.84502800
H	−4.06698300	−1.41439100	−0.09851400
H	−5.27114600	−2.68045300	0.12231800
H	−4.88129200	−2.26827800	−3.96372000
H	−3.62871100	−1.02658200	−4.24376200
H	−2.71318000	−2.94348700	−3.17870200

H	−2.56679900	−1.58770200	−2.21491900
H	−4.54678000	−3.82275500	−1.94451900
H	−3.11848700	−3.49654000	−0.90312600
H	−4.31512300	0.19085100	−2.20754000
N	−3.26570200	−2.29588300	−2.61244100

**NAME:**	unsolvated [Ru]-H + triethylamine-H+
**SINGLE−POINT ENERGY (DFT):**	−2247.01336991 hartree
**CHARGE, MULTIPLICITY:**	1, 1
**STRUCTURE TYPE:**	MINIMUM
**COMMENT:**	…

C	1.37570900	−0.31452000	−0.85568000
H	1.09692900	−0.24666000	−1.92481100
C	1.88995500	−1.74144800	−0.61196200
H	2.21048500	−1.80093200	0.43903100
N	0.74128200	−2.66121000	−0.74269800
H	0.42075600	−2.68182200	−1.71068600
H	1.04027700	−3.61291900	−0.52860400
N	0.18603200	−0.12308800	−0.01094700
C	3.05892200	−2.10505800	−1.49966700
C	4.33564200	−2.24371100	−0.95071100
C	2.89369900	−2.26553000	−2.88008200
C	5.42850200	−2.54239200	−1.76230500
H	4.47920900	−2.09998200	0.12320400
C	3.98286000	−2.56970400	−3.69320400
H	1.90844600	−2.14099800	−3.34038300
C	5.25330400	−2.70866700	−3.13450400
H	6.42106600	−2.64546200	−1.31957100
H	3.84061300	−2.69534800	−4.76835700
H	6.10773700	−2.94573900	−3.77154000
C	2.50313900	0.67337100	−0.60589000
C	3.19895400	1.23433300	−1.67729100
C	2.88764500	0.99706300	0.69767900
C	4.25682400	2.11432900	−1.45276000
H	2.90981200	0.98085000	−2.70055800
C	3.93854500	1.88014500	0.92628000
H	2.33652000	0.57091500	1.53797000
C	4.62603600	2.44270200	−0.15013700
H	4.79173400	2.54751200	−2.30039300
H	4.22051400	2.13467300	1.95028700
H	5.45039500	3.13653900	0.02801100
S	−0.68596200	1.16731700	−0.41561400
O	−0.83235200	1.34981300	−1.87155000
O	−1.97834700	1.11542600	0.32535600
C	0.14730200	2.60438300	0.21353300
C	0.15228800	2.83360100	1.58766700
C	0.83995800	3.44080800	−0.65636500

C	0.88470500	3.90116000	2.09403100
H	−0.41314700	2.18170300	2.25610900
C	1.57211200	4.50049200	−0.13320800
H	0.81718200	3.24577400	−1.72899300
C	1.61895400	4.74039200	1.24583000
H	0.89290300	4.08348900	3.17127700
H	2.13298700	5.14828500	−0.81100700
C	2.45973700	5.86114900	1.79214900
H	2.25424400	6.80601800	1.26799000
H	2.28341000	6.01869300	2.86453300
H	3.52954300	5.63841500	1.65361100
Ru	−0.83749200	−1.94217700	0.50979500
H	−4.03926900	−3.39640000	−0.15608700
C	−3.45270100	−4.02792700	0.52483100
C	−2.43018100	−3.21506200	1.26464900
H	−2.97663200	−4.82057400	−0.06759300
H	−4.14773000	−4.50214500	1.23508400
C	−1.17795600	−3.75932100	1.64140900
C	−2.68137700	−1.85110200	1.62792300
C	−0.22482600	−3.02284500	2.42611400
H	−0.93580200	−4.77972600	1.33501100
C	−1.69676400	−1.10803400	2.30804700
H	−3.60313400	−1.35934100	1.31565700
C	−0.46282600	−1.68892700	2.76809000
H	0.72260700	−3.49822400	2.68276200
H	−1.87031800	−0.04227700	2.45805800
C	0.52975600	−0.79481500	3.48033100
H	0.60384300	0.10629700	2.84602300
C	1.92726000	−1.39336000	3.60255300
C	−0.01292700	−0.36941300	4.84978200
H	2.32988100	−1.69642700	2.62425000
H	2.62039000	−0.65403700	4.02901200
H	1.93737500	−2.27238800	4.26608600
H	0.66941800	0.34842100	5.32836500
H	−1.00038500	0.10839100	4.76746200
H	−0.11424600	−1.23831100	5.51836000
H	−1.59821500	−1.78472600	−0.88675400
N	−4.01027500	1.10145300	−1.42775700
C	−3.74207700	−0.11255100	−2.26282900
H	−2.73497600	0.04428300	−2.67300100
H	−3.65556100	−0.94945400	−1.55477800
C	−4.75180000	−0.41157700	−3.35242100
H	−4.37514000	−1.25593600	−3.94589600
H	−5.73016700	−0.70852900	−2.95071200
H	−4.89186500	0.43267900	−4.04239000
C	−5.24670200	1.02964800	−0.58587300
H	−5.02914000	1.60141800	0.32756200
H	−5.34697500	−0.02290600	−0.28506900
C	−6.52882400	1.53320300	−1.22545500
H	−6.77385200	1.01358500	−2.15975800
H	−7.35434100	1.36014300	−0.52132500

H	−6.49583600	2.61217900	−1.42742700
C	−3.82470200	2.38355800	−2.17519100
H	−2.87782500	2.26461600	−2.71744000
H	−4.63993100	2.48494600	−2.90420400
C	−3.73197500	3.58152900	−1.24799800
H	−2.93738500	3.43389500	−0.50271800
H	−3.47965900	4.47105200	−1.84053500
H	−4.67435900	3.79030800	−0.72221000
H	−3.21805000	1.08750300	−0.72888900

**NAME:**	unsolvated [Ru]-H + tributylamine-H+
**SINGLE−POINT ENERGY (DFT):**	−2482.67048397 hartree
**CHARGE, MULTIPLICITY:**	1, 1
**STRUCTURE TYPE:**	MINIMUM
**COMMENT:**	…

C	−1.96859300	−0.14670700	0.85664700
H	−1.51107000	−0.09530400	1.86343800
C	−2.74336200	−1.47263900	0.78388700
H	−3.23576500	−1.51315300	−0.19932200
N	−1.75555900	−2.56941500	0.79470200
H	−1.29100700	−2.61123300	1.70153500
H	−2.23590500	−3.46292100	0.68581400
N	−0.91823200	−0.18901600	−0.17353000
C	−3.79589200	−1.59557200	1.86293900
C	−5.14775600	−1.46408000	1.53673000
C	−3.43814700	−1.78367300	3.20261900
C	−6.12583400	−1.52162300	2.52731900
H	−5.43588400	−1.29558600	0.49596900
C	−4.41398100	−1.84884400	4.19453000
H	−2.38561200	−1.86944600	3.49047000
C	−5.76097500	−1.71633400	3.85816300
H	−7.17818400	−1.41067500	2.25896800
H	−4.12249200	−1.99831200	5.23607000
H	−6.52624100	−1.76310500	4.63527000
C	−2.94798200	1.00816700	0.72862500
C	−3.39860600	1.67584800	1.86824300
C	−3.45221800	1.37890900	−0.52016800
C	−4.33562100	2.70234500	1.76414900
H	−3.01372100	1.38665200	2.84965800
C	−4.38266600	2.40832100	−0.62919500
H	−3.08648900	0.86990100	−1.41381500
C	−4.82747400	3.07326200	0.51429000
H	−4.68230300	3.21465500	2.66394400
H	−4.76196500	2.69638400	−1.61210500
H	−5.56092400	3.87806500	0.43051500
S	0.22141600	0.92890900	0.03697400
O	0.64611200	1.08129900	1.44066800

O	1.33232900	0.65388200	−0.91694900
C	−0.44582200	2.49413500	−0.47414100
C	−0.62959400	2.74072600	−1.83300400
C	−0.84127200	3.42260400	0.48350800
C	−1.24490600	3.92232700	−2.22941900
H	−0.29426900	2.01251600	−2.57381700
C	−1.46032000	4.59734000	0.07062200
H	−0.68271200	3.21112900	1.54148800
C	−1.68641600	4.85986400	−1.28592200
H	−1.39385400	4.11878500	−3.29384500
H	−1.78802700	5.32055900	0.82093600
C	−2.41900100	6.10297200	−1.70960700
H	−2.10004000	6.97742300	−1.12464800
H	−2.26415100	6.32375300	−2.77432900
H	−3.50152100	5.97693200	−1.54654800
Ru	−0.29633900	−2.17836300	−0.71798600
H	2.62872900	−4.19058900	−0.36679600
C	1.85503100	−4.72480800	−0.93500000
C	0.90771800	−3.76042500	−1.58834800
H	1.32496100	−5.39396100	−0.24422700
H	2.35445500	−5.34425100	−1.69599400
C	−0.46485200	−4.06507500	−1.76279400
C	1.35497300	−2.48791900	−2.07450400
C	−1.36618200	−3.19089400	−2.46279600
H	−0.84909000	−5.00400700	−1.35659300
C	0.44187900	−1.59555800	−2.66839900
H	2.38772700	−2.17313000	−1.91990900
C	−0.93346500	−1.94102000	−2.91436500
H	−2.41196100	−3.48373700	−2.56282500
H	0.78755000	−0.59085700	−2.91214300
C	−1.83388100	−0.89986300	−3.54442400
H	−1.62049100	0.03206300	−2.99138200
C	−3.32289200	−1.19477400	−3.39504800
C	−1.45571900	−0.68353200	−5.01448500
H	−3.60307800	−1.34968500	−2.34251100
H	−3.91604500	−0.35062900	−3.77496400
H	−3.62164500	−2.08757300	−3.96666600
H	−2.04761100	0.13489500	−5.44998100
H	−0.39176600	−0.42899900	−5.12970400
H	−1.64850200	−1.59327000	−5.60402700
H	0.68190000	−2.10032800	0.54371300
N	3.64242500	0.41659100	0.43763300
C	3.36405500	−0.83001500	1.21475400
H	2.43071800	−0.62359000	1.75838700
H	3.11650900	−1.60174200	0.47093100
C	4.45302700	−1.31260600	2.15434300
H	5.35220100	−1.60449900	1.58788300
H	4.75580400	−0.50590000	2.84236900
C	4.73442500	0.30412900	−0.57762600
H	4.45532800	0.96802500	−1.40908400
H	4.69072900	−0.72516300	−0.96345800

C	6.14060700	0.63770700	−0.10100100
H	6.38218700	0.08919200	0.82162500
H	6.21016800	1.70916000	0.14540900
C	3.69432700	1.64724600	1.28294300
H	2.85575600	1.55665800	1.98581900
H	4.63480100	1.64436300	1.84882300
C	3.51722200	2.91193600	0.45105300
H	2.70438300	2.74444800	−0.27329200
H	4.42902600	3.12013200	−0.13426700
H	2.75958800	0.52299000	−0.12854500
C	3.16996100	4.12901000	1.30874000
H	2.20756500	3.93989800	1.81319500
C	7.17806800	0.30685600	−1.17412900
H	7.12947200	−0.77127400	−1.40452000
H	6.91608100	0.83034800	−2.10947100
C	8.59604700	0.67862100	−0.75778500
H	9.32111300	0.41473500	−1.54012200
H	8.68707800	1.75929500	−0.56905800
H	8.89164200	0.15247700	0.16302800
C	3.96789400	−2.51371600	2.96694700
H	3.64276300	−3.30935800	2.27455600
H	3.06870800	−2.22340800	3.53612900
C	5.02817000	−3.05719500	3.91662200
H	4.64901800	−3.91868400	4.48397600
H	5.92486400	−3.38692200	3.36930100
H	5.34300700	−2.29181400	4.64245700
H	2.99518700	4.98077300	0.63344300
C	4.23677500	4.50031000	2.33403600
H	4.36292100	3.72134100	3.10254600
H	5.21509100	4.65855200	1.85235000
H	3.97308700	5.42929400	2.85906600

**NAME:**	IEFPCM [Ru]-H
**SINGLE−POINT ENERGY (DFT):**	−1954.45049187 hartree
**CHARGE, MULTIPLICITY:**	0, 1
**STRUCTURE TYPE:**	MINIMUM
**COMMENT:**	…

C	−0.46563600	1.00580200	−0.65785500
H	−0.53878600	1.42878300	−1.67810500
C	0.35171500	2.00080900	0.18398600
H	0.40086300	1.60508600	1.20924500
N	1.73380100	1.98803800	−0.32579700
H	1.77014400	2.39124100	−1.26203200
H	2.32367800	2.58196000	0.25782500
N	0.26968100	−0.26373800	−0.68139300
C	−0.26996500	3.38015200	0.22644000
C	−0.83257300	3.85700100	1.41347100
C	−0.33594200	4.17826400	−0.92174200
C	−1.45453900	5.10455400	1.45559700
H	−0.79394800	3.23726200	2.31312100
C	−0.95196600	5.42774600	−0.88215600
H	0.08546000	3.82424000	−1.86693600
C	−1.51497000	5.89318600	0.30700400
H	−1.89387000	5.46189600	2.38941000
H	−0.99709600	6.03858500	−1.78639200
H	−2.00163800	6.87035400	0.33740600
C	−1.87593300	0.92498800	−0.09330100
C	−2.92710500	1.59825000	−0.71822600
C	−2.13192100	0.21914700	1.08521500
C	−4.21332800	1.56648600	−0.17908700
H	−2.73718600	2.15078900	−1.64234200
C	−3.41442700	0.17930200	1.62467800
H	−1.31694200	−0.32424800	1.56567500
C	−4.46094300	0.85507400	0.99383900
H	−5.02702300	2.09445200	−0.68177800
H	−3.60079600	−0.38572500	2.54088300
H	−5.46803700	0.82219400	1.41577400
S	−0.25045600	−1.29932800	−1.82162900
O	−0.62980700	−0.60805100	−3.06700000
O	0.69729400	−2.41115300	−1.94998400
C	−1.75045100	−2.03046900	−1.17625300
C	−1.65998200	−2.95337500	−0.13655700
C	−2.99134300	−1.64448700	−1.67326700
C	−2.82450300	−3.46051400	0.43207600
H	−0.68278700	−3.27250300	0.23069500
C	−4.14856200	−2.15843800	−1.09689700
H	−3.04191600	−0.92683000	−2.49255800
C	−4.08616600	−3.05998400	−0.02695800
H	−2.75210600	−4.17927000	1.25226600

H	−5.12255700	−1.84088600	−1.47802900
C	−5.34871500	−3.56015800	0.62174600
H	−6.07084600	−3.91428200	−0.12871200
H	−5.14644100	−4.38089900	1.32325900
H	−5.83698900	−2.74803300	1.18410700
Ru	2.38874700	−0.04598300	−0.38102000
H	5.44514900	−0.44168400	−2.20858900
C	5.64740000	−0.16156000	−1.16620200
C	4.50446400	−0.55119700	−0.27344300
H	5.82808000	0.92112700	−1.12453300
H	6.56856100	−0.67408200	−0.84777700
C	4.14531900	0.22417900	0.85565900
C	3.73192700	−1.73409700	−0.52242300
C	3.11743300	−0.18264800	1.77367200
H	4.66973000	1.16517300	1.03728100
C	2.65934700	−2.07262600	0.32615800
H	3.92295200	−2.33339700	−1.41348800
C	2.35374900	−1.32658600	1.52083600
H	2.88803200	0.46607300	2.61931800
H	2.01368200	−2.90116900	0.03582600
C	1.14916000	−1.74099200	2.33958100
H	0.32892400	−1.80761900	1.60400800
C	0.73695500	−0.72243800	3.39763000
C	1.35409400	−3.12749100	2.95882800
H	0.56050400	0.27193200	2.96116800
H	−0.19629600	−1.04216400	3.88383100
H	1.50296800	−0.61886900	4.18240800
H	0.43600300	−3.46013100	3.46580900
H	1.60937500	−3.88099800	2.19934500
H	2.16598000	−3.10707700	3.70303200
H	2.48778200	0.23749200	−1.95556500

**NAME:**	IEFPCM butylamine-H+
**SINGLE−POINT ENERGY (DFT):**	−214.05032920 hartree
**CHARGE, MULTIPLICITY:**	1, 1
**STRUCTURE TYPE:**	MINIMUM
**COMMENT:**	…

C	−2.58323000	−0.32121800	−0.00702400
H	−2.63493100	−0.92624500	−0.92564300
H	−2.62062300	−1.01092700	0.85049200
H	−3.48195200	0.31119100	0.02974900
C	−1.31564400	0.52466700	0.02193200
H	−1.31021400	1.21652300	−0.83676200
H	−1.30932800	1.15591100	0.92626700
C	−0.04508400	−0.32339500	−0.00495900
H	−0.02593400	−0.98672900	0.87633900

H	−0.05384000	−0.97500400	−0.89528700
C	1.20515700	0.53736100	−0.02259900
H	1.26218000	1.14902500	−0.93194100
H	1.25299200	1.20608100	0.84623700
N	2.44097200	−0.29954900	0.01109600
H	2.46482700	−0.97245000	−0.76368300
H	3.29321100	0.26761700	−0.05698500
H	2.50961100	−0.84264000	0.87944600

**NAME:**	IEFPCM piperidine-H+
**SINGLE−POINT ENERGY (DFT):**	−252.12275326 hartree
**CHARGE, MULTIPLICITY:**	1, 1
**STRUCTURE TYPE:**	MINIMUM
**COMMENT:**	…

C	−0.74584500	1.25955000	−0.22499900
C	−1.48909700	0.00057300	0.22224700
C	−0.74683500	−1.25899000	−0.22498700
C	0.70023200	−1.25134900	0.23919200
C	0.70121000	1.25082300	0.23920800
H	−1.22428600	−2.16317200	0.17743000
H	−1.58410700	0.00061400	1.32085200
H	−2.50897400	0.00097400	−0.18685700
H	−0.77625500	1.35038200	−1.32349100
H	−1.22263300	2.16411000	0.17735500
H	0.77453500	−1.25751100	1.33518300
H	1.27192700	−2.09971600	−0.15491100
H	2.35883500	−0.00092900	0.09564900
H	1.43398800	−0.00052700	−1.24843600
H	0.77552500	1.25693100	1.33519800
H	1.27355200	2.09875400	−0.15489600
H	−0.77735900	−1.34985700	−1.32347200
N	1.38389500	−0.00052700	−0.22193900

**NAME:**	IEFPCM triethylamine-H+
**SINGLE−POINT ENERGY (DFT):**	−292.58658666 hartree
**CHARGE, MULTIPLICITY:**	1, 1
**STRUCTURE TYPE:**	MINIMUM
**COMMENT:**	…

N	0.06246100	−0.20392700	0.66178100
C	−1.39186700	−0.62209100	0.69241000
H	−1.41535700	−1.57285800	1.24141100
H	−1.90705100	0.12779400	1.30592900
C	−2.06351800	−0.79127800	−0.65709600

H	−3.12220100	−1.01437000	−0.46770500
H	−2.02195700	0.11218800	−1.27668800
H	−1.64878800	−1.63284000	−1.22665500
C	0.28077000	1.28776400	0.65043500
H	1.34705000	1.43833800	0.85851200
H	−0.27415500	1.67509500	1.51431700
C	−0.12548100	2.00200500	−0.62080200
H	−1.21157700	1.99593200	−0.77584500
H	0.18541300	3.05073900	−0.52296600
H	0.36943000	1.59442200	−1.51290300
C	0.86078200	−0.96176200	−0.35972700
H	0.60293900	−2.01910700	−0.21354400
H	0.49777900	−0.66278300	−1.35009900
C	2.35519900	−0.75102600	−0.22949700
H	2.71232400	−0.97641900	0.78635100
H	2.85821200	−1.44058000	−0.92005700
H	2.66298200	0.26807000	−0.49902600
H	0.43242900	−0.49780000	1.57216700

**NAME:**	IEFPCM tributylamine-H+
**SINGLE−POINT ENERGY (DFT):**	−528.23707143 hartree
**CHARGE, MULTIPLICITY:**	1, 1
**STRUCTURE TYPE:**	MINIMUM
**COMMENT:**	…

C	4.36837500	0.64436800	−0.46679000
H	3.73453800	1.46540700	−0.83570900
H	4.68415800	0.89717000	0.55774600
H	5.26791000	0.62170300	−1.09882600
C	3.64983100	−0.70158300	−0.50307600
H	3.31290800	−0.91912500	−1.53057600
H	4.36039700	−1.50014500	−0.24074900
C	2.45596900	−0.81429900	0.44861900
H	2.06902000	−1.84345100	0.40601600
H	2.79149600	−0.64224700	1.48556400
C	1.35160600	0.17324000	0.10701400
H	1.69907900	1.20710300	0.23015900
H	1.01205900	0.05556200	−0.92924500
N	0.15540200	0.03403800	1.00180700
H	0.52612400	0.05171300	1.95762900
C	−0.75487300	1.23284600	0.94934200
H	−1.46813400	1.11130600	1.77608500
H	−0.11485800	2.09290100	1.18629100
C	−1.49105300	1.44257300	−0.36291600
H	−0.78264200	1.44412100	−1.20689600
H	−2.19723900	0.61613100	−0.52792300
C	−2.27527700	2.75956400	−0.36611500
H	−2.89715900	2.76987200	−1.27432700

H	−2.97944300	2.76654800	0.48280700
C	−1.41037700	4.01681600	−0.33635200
H	−2.03138300	4.91739600	−0.44716200
H	−0.85649500	4.12290000	0.60895400
H	−0.67627400	4.01204000	−1.15732300
C	−0.56464000	−1.29082400	0.89656500
H	−0.00495200	−2.00064500	1.52009500
H	−1.53597700	−1.13391000	1.38224900
C	−0.72396900	−1.85524100	−0.50860300
H	−1.13239800	−1.10179600	−1.19738100
H	0.25998200	−2.14423000	−0.90599700
C	−1.63591200	−3.08687500	−0.51159200
H	−1.53564800	−3.57245600	−1.49413000
H	−1.26427400	−3.81775000	0.22636100
C	−3.10895800	−2.78465200	−0.25067100
H	−3.50233200	−2.07091500	−0.99159100
H	−3.71119200	−3.70227000	−0.31654200
H	−3.27941700	−2.35680300	0.74923200

**NAME:**	IEFPCM [Ru]-H + butylamine-H+
**SINGLE−POINT ENERGY (DFT):**	−2168.53183188 hartree
**CHARGE, MULTIPLICITY:**	1, 1
**STRUCTURE TYPE:**	MINIMUM
**COMMENT:**	…

C	0.82242600	1.04741200	−0.74589900
H	0.67791900	1.12019500	−1.84068800
C	2.33728700	1.11560300	−0.48402000
H	2.48428100	1.08342100	0.60524900
N	2.94375600	−0.11676900	−1.01689200
H	2.88850400	−0.12938700	−2.03536900
H	3.93887900	−0.13691600	−0.79167400
N	0.35107300	−0.25688900	−0.25581700
C	2.96115200	2.38804200	−1.01527300
C	3.41309500	3.36549600	−0.12432300
C	3.05330200	2.63053100	−2.39049600
C	3.94409300	4.56587100	−0.59520000
H	3.33569600	3.18896200	0.95158400
C	3.58906500	3.82676500	−2.86452600
H	2.69627700	1.88830400	−3.11017900
C	4.03415400	4.79853600	−1.96715000
H	4.28896700	5.32155900	0.11371700
H	3.65624200	4.00248600	−3.94020300
H	4.45133800	5.73685800	−2.33900300
C	0.15291900	2.25385800	−0.10760900
C	−0.21312700	3.35138000	−0.88876900
C	−0.06199700	2.30208600	1.27216000
C	−0.79112600	4.47816400	−0.30439400

H	−0.04698900	3.32199800	−1.96892600
C	−0.64578000	3.42108800	1.85921700
H	0.20672200	1.43833000	1.88270400
C	−1.01255700	4.51411100	1.07149700
H	−1.07675200	5.32793500	−0.92849400
H	−0.82110700	3.43946300	2.93721700
H	−1.47337900	5.39133600	1.53108600
S	−1.11888000	−0.66134800	−0.75812000
O	−1.35858900	−0.35102900	−2.19068800
O	−1.39602200	−2.06898800	−0.39165400
C	−2.29544600	0.30762600	0.16475800
C	−2.54701700	−0.03314300	1.49123600
C	−2.92228700	1.40154900	−0.42400400
C	−3.42348200	0.74742300	2.23861900
H	−2.06406500	−0.90377300	1.93866600
C	−3.79189500	2.17453100	0.33618100
H	−2.72133300	1.64590100	−1.46731800
C	−4.05014300	1.86782800	1.67828400
H	−3.61998900	0.48402800	3.28059600
H	−4.27744400	3.03962400	−0.12184600
C	−4.96626900	2.73814300	2.49440800
H	−5.92620900	2.89551200	1.98057900
H	−5.16917000	2.29857300	3.48016100
H	−4.51359700	3.73011400	2.65060800
Ru	1.87878400	−1.78098300	−0.19529400
H	2.58648900	−4.90927600	−1.80883800
C	3.34192600	−4.67440800	−1.04740300
C	2.78313000	−3.75208300	−0.00268000
H	4.21353900	−4.22710900	−1.54384100
H	3.66200100	−5.61927500	−0.58146700
C	3.59331600	−2.79174300	0.65107600
C	1.40283500	−3.81067900	0.38296700
C	3.09931300	−1.96663000	1.71899200
H	4.63296100	−2.67685800	0.33611500
C	0.90518000	−2.92617200	1.35975000
H	0.71808400	−4.48616500	−0.13089600
C	1.75099200	−2.01517600	2.08669100
H	3.77709400	−1.24405100	2.17431500
H	−0.16939200	−2.91303000	1.54297200
C	1.11026400	−1.10413700	3.11261700
H	0.23885500	−0.66243700	2.59809600
C	2.00663500	0.03935200	3.57663500
C	0.59426900	−1.91902200	4.30438300
H	2.38045400	0.63685800	2.73223300
H	1.44250400	0.71347900	4.23730300
H	2.87556600	−0.33128100	4.14258600
H	0.06007000	−1.26769500	5.01188700
H	−0.10022800	−2.71032400	3.98597400
H	1.43030000	−2.39483400	4.84093700
H	1.51970200	−2.05975400	−1.73413300
C	−6.40028200	−0.38316100	−0.57914700

H	−5.90076900	−0.47266000	0.39892200
H	−6.08796300	0.57786100	−1.01634300
H	−7.48537900	−0.34080100	−0.40563500
C	−6.02687800	−1.54569300	−1.49282500
H	−6.39650600	−2.49260900	−1.06441200
H	−6.52898300	−1.43098400	−2.46830900
C	−4.51980300	−1.63889700	−1.70241800
H	−4.14261700	−0.68729200	−2.11173400
H	−4.02245100	−1.77242100	−0.72855000
C	−4.11021500	−2.77233800	−2.62669000
H	−4.43053700	−3.74804800	−2.23742900
H	−4.53378800	−2.64892500	−3.63214100
N	−2.63085400	−2.81689600	−2.76521800
H	−2.16974200	−2.93010200	−1.83886900
H	−2.31199800	−3.55320800	−3.39940200
H	−2.24972900	−1.90741100	−3.08059900

**NAME:**	IEFPCM [Ru]-H + piperidine-H+ (1)
**SINGLE−POINT ENERGY (DFT):**	−2206.60428599 hartree
**CHARGE, MULTIPLICITY:**	1, 1
**STRUCTURE TYPE:**	MINIMUM
**COMMENT:**	…

C	0.96946400	−0.52392700	−0.85911500
H	0.58814900	−0.36830500	−1.88651600
C	1.01123700	−2.04059500	−0.60902400
H	1.41337400	−2.19250500	0.40287500
N	−0.37623900	−2.53624200	−0.57835900
H	−0.78454100	−2.50379900	−1.51285200
H	−0.38342400	−3.52050800	−0.30780300
N	0.03874900	0.05361300	0.12153900
C	1.89547700	−2.77901600	−1.59028600
C	3.06924400	−3.39192800	−1.14351500
C	1.57995200	−2.83187100	−2.95304800
C	3.91668300	−4.04341500	−2.03905500
H	3.32718900	−3.34904000	−0.08213200
C	2.42218600	−3.48635400	−3.84987900
H	0.67406300	−2.34997200	−3.33175700
C	3.59441500	−4.09200700	−3.39478100
H	4.83218000	−4.51442400	−1.67502500
H	2.16526900	−3.51939700	−4.91078800
H	4.25610400	−4.60204700	−4.09807300
C	2.38851200	0.01751600	−0.79954200
C	3.07693200	0.32408400	−1.97434900
C	3.04127700	0.16370400	0.42742700
C	4.39522100	0.77747100	−1.92692700
H	2.57427700	0.21015800	−2.93841100
C	4.35389900	0.62402700	0.48014200

H	2.50144100	−0.06068800	1.34965400
C	5.03559800	0.93244300	−0.69874600
H	4.92046000	1.01725700	−2.85413200
H	4.84678300	0.74947600	1.44676700
H	6.06424800	1.29772200	−0.65750700
S	−0.37923000	1.58681100	−0.16862700
O	−0.65513600	1.83195300	−1.61173900
O	−1.47637800	1.97243700	0.72816600
C	1.00482800	2.61624100	0.26221800
C	1.28821800	2.80820400	1.61389400
C	1.81025000	3.17538600	−0.72363200
C	2.41078100	3.54410700	1.97405700
H	0.63548500	2.38440200	2.37957200
C	2.93407300	3.90548400	−0.34741700
H	1.56832500	3.02212200	−1.77560400
C	3.26115300	4.08895100	1.00120300
H	2.63538400	3.69383600	3.03286300
H	3.57731000	4.33120400	−1.12121000
C	4.51573800	4.81921700	1.39528800
H	4.78486000	5.58653800	0.65631800
H	4.41256700	5.29874900	2.37857000
H	5.35871500	4.11166800	1.45781900
Ru	−1.50604400	−1.31444900	0.75601300
H	−5.03970700	−1.59967100	0.37699700
C	−4.66366000	−2.39481000	1.03484200
C	−3.36620100	−1.99317700	1.67454700
H	−4.54499000	−3.31223800	0.44335600
H	−5.42199100	−2.58253800	1.81051800
C	−2.34069700	−2.93389100	1.93215600
C	−3.11435300	−0.63103200	2.04708700
C	−1.11661600	−2.56779400	2.58956200
H	−2.47758100	−3.96758900	1.60803300
C	−1.88041700	−0.27247600	2.62384000
H	−3.85077800	0.14359100	1.82911000
C	−0.86371700	−1.23736500	2.94522600
H	−0.35465000	−3.33458500	2.73380200
H	−1.67755800	0.78562000	2.78555300
C	0.44588700	−0.73620200	3.51837000
H	0.75322200	0.08014400	2.84162100
C	1.56026400	−1.77722500	3.52344400
C	0.23549100	−0.14396100	4.91615500
H	1.73065900	−2.20168900	2.52317600
H	2.50237000	−1.31660900	3.85408300
H	1.33655300	−2.60738400	4.21174400
H	1.17202000	0.29497300	5.29090400
H	−0.52856400	0.64706100	4.91330400
H	−0.08437200	−0.92281400	5.62621600
H	−2.27929900	−0.95755200	−0.60980200
C	−4.47455200	3.36786800	−1.92638000
C	−5.11339600	2.78168300	−3.18613300
C	−4.26517600	1.63876500	−3.74741800

C	−3.99300900	0.58035500	−2.68925900
C	−4.20634700	2.28978000	−0.88811400
H	−4.76380700	1.15849800	−4.60115100
H	−6.12117600	2.40416700	−2.94317700
H	−5.24202300	3.56490800	−3.94691300
H	−3.52286800	3.86471100	−2.18059300
H	−5.12134800	4.13371700	−1.47555400
H	−4.92096400	0.08889600	−2.36255200
H	−3.29860300	−0.19346200	−3.04187600
H	−3.16063000	0.47324900	−0.78944200
H	−2.43654400	1.57712800	−1.72613500
H	−5.14021000	1.82923900	−0.53379300
H	−3.64400900	2.66581100	−0.02555600
H	−3.30468600	2.03210000	−4.12095300
N	−3.38433900	1.20721200	−1.48868800

**NAME:**	IEFPCM [Ru]-H + piperidine-H+ (2)
**SINGLE−POINT ENERGY (DFT):**	−2206.60265900 hartree
**CHARGE, MULTIPLICITY:**	1, 1
**STRUCTURE TYPE:**	MINIMUM
**COMMENT:**	…

C	0.71055300	1.02930100	−0.71578500
H	0.38008400	1.04091800	−1.77195800
C	2.24567300	1.13583800	−0.72140500
H	2.57742500	1.15431600	0.32722400
N	2.78117400	−0.10869900	−1.30012000
H	2.55314300	−0.16494100	−2.29288900
H	3.80039300	−0.10081200	−1.24890100
N	0.36381100	−0.25532900	−0.08908800
C	2.74443000	2.38953500	−1.40686600
C	3.36679500	3.39287600	−0.65937300
C	2.55880700	2.58500800	−2.78038100
C	3.79317500	4.57264800	−1.26860500
H	3.51063600	3.25179000	0.41486300
C	2.98765600	3.76077700	−3.39313400
H	2.06445200	1.82065500	−3.38678700
C	3.60423500	4.75909700	−2.63746800
H	4.27494700	5.34922600	−0.67070900
H	2.83454300	3.90102800	−4.46518400
H	3.93750300	5.68204300	−3.11673500
C	0.13164000	2.25792700	−0.03296200
C	−0.38270200	3.30950800	−0.79356100
C	0.14830400	2.37356800	1.35944500
C	−0.87918600	4.45698600	−0.17567100
H	−0.40098600	3.22646600	−1.88350100
C	−0.35449300	3.51311700	1.98137300
H	0.53376900	1.54621500	1.95764000

C	−0.86961300	4.56012800	1.21455700
H	−1.28240000	5.27006200	−0.78341600
H	−0.34930700	3.58386400	3.07140600
H	−1.26732200	5.45323000	1.70164100
S	−1.16046500	−0.71832500	−0.30135300
O	−1.63584300	−0.46920100	−1.69654200
O	−1.35559700	−2.09434500	0.16712600
C	−2.19162000	0.30116300	0.73577800
C	−2.23994400	0.02386600	2.10012300
C	−2.91136300	1.36407500	0.19768200
C	−2.99834900	0.84020700	2.93307500
H	−1.69301000	−0.82742700	2.50958400
C	−3.66519900	2.17131500	1.04198200
H	−2.86250900	1.56701200	−0.87240300
C	−3.71231800	1.93170300	2.42149000
H	−3.03575600	0.62554700	4.00378200
H	−4.21983900	3.01333200	0.62100300
C	−4.49439300	2.84463200	3.32575300
H	−5.47971300	3.07961000	2.89796600
H	−4.64225600	2.40084000	4.31951100
H	−3.96053700	3.79935800	3.45843600
Ru	1.91157300	−1.75309100	−0.24962600
H	2.54067700	−4.87493200	−1.90777200
C	3.38818800	−4.57801600	−1.27563700
C	2.93963200	−3.66849500	−0.16824800
H	4.14078700	−4.08752500	−1.90763800
H	3.83720800	−5.49142400	−0.85556700
C	3.78248100	−2.65264300	0.34650500
C	1.63873600	−3.79356500	0.42299700
C	3.40984700	−1.83464400	1.46719500
H	4.75451800	−2.48836700	−0.12428600
C	1.24038200	−2.92001500	1.45301600
H	0.92525900	−4.51727500	0.02656800
C	2.13172500	−1.94277900	2.02471800
H	4.10712800	−1.07020500	1.81029000
H	0.20597400	−2.95981200	1.79381100
C	1.59117800	−1.02182500	3.09887300
H	0.65372800	−0.61916600	2.67657800
C	2.50034700	0.16066200	3.41770900
C	1.24468700	−1.80973900	4.36713300
H	2.74686200	0.74198100	2.51679900
H	2.00089400	0.83887400	4.12477000
H	3.44413600	−0.16710700	3.88115000
H	0.78066700	−1.14794400	5.11345700
H	0.53969100	−2.62809800	4.15994400
H	2.15139200	−2.24635400	4.81486400
H	1.29460100	−2.09368000	−1.68916900
C	−4.78218500	−2.05692700	−0.57788700
C	−5.79009600	−1.20642200	−1.34932900
C	−5.11474300	−0.50998100	−2.52994700
C	−4.40852700	−1.51045900	−3.43117900

C	−4.04231300	−3.02836500	−1.48459500
H	−5.84448800	0.04568800	−3.13531000
H	−6.60998600	−1.84533700	−1.71900600
H	−6.24194800	−0.46124700	−0.67887300
H	−4.04823400	−1.40847500	−0.07630300
H	−5.27683300	−2.63871800	0.21273300
H	−5.11886400	−2.22109700	−3.87771700
H	−3.85380700	−1.01870400	−4.23983700
H	−2.94482100	−2.96947300	−3.24329400
H	−2.68818400	−1.64313100	−2.27654500
H	−4.72086900	−3.78493500	−1.90398600
H	−3.22615600	−3.53143100	−0.95229500
H	−4.37746200	0.22193000	−2.16243200
N	−3.42841000	−2.30299200	−2.63582300

**NAME:**	IEFPCM [Ru]-H + triethylamine-H+
**SINGLE−POINT ENERGY (DFT):**	−2247.07034617 hartree
**CHARGE, MULTIPLICITY:**	1, 1
**STRUCTURE TYPE:**	MINIMUM
**COMMENT:**	…

C	1.35432900	−0.36011200	−0.88535300
H	1.05566700	−0.34092000	−1.95060200
C	1.84733200	−1.78330900	−0.57850700
H	2.17960400	−1.80000100	0.46966900
N	0.68718600	−2.68945300	−0.65535400
H	0.36641500	−2.77327900	−1.62014400
H	0.96554400	−3.62953200	−0.37183100
N	0.18795900	−0.10445100	−0.02779700
C	3.00717600	−2.20003300	−1.45694700
C	4.27729100	−2.37181900	−0.89981400
C	2.84130500	−2.38229900	−2.83481300
C	5.36454900	−2.71774800	−1.70185100
H	4.41846900	−2.22321500	0.17381500
C	3.92464900	−2.73310800	−3.63825700
H	1.86053000	−2.24228600	−3.29839900
C	5.18979600	−2.90015800	−3.07312300
H	6.35142100	−2.84618200	−1.25217200
H	3.78066500	−2.87326300	−4.71157400
H	6.03921400	−3.17277700	−3.70321900
C	2.50019700	0.62225100	−0.70826200
C	3.14156400	1.16788000	−1.82122000
C	2.95221600	0.96352200	0.56976300
C	4.21346100	2.04692900	−1.66355000
H	2.79346200	0.90731400	−2.82431100
C	4.01766100	1.84416400	0.73236500
H	2.44448700	0.55142100	1.44390400
C	4.65219400	2.38945900	−0.38574900

H	4.70180300	2.47133600	−2.54354000
H	4.35196100	2.11178800	1.73725300
H	5.48448600	3.08546200	−0.25906200
S	−0.65257000	1.20233300	−0.44466600
O	−0.76736700	1.38376500	−1.90526200
O	−1.94443300	1.19213400	0.28718800
C	0.21724400	2.62318700	0.18462200
C	0.25604900	2.83340600	1.56148600
C	0.89589100	3.46935900	−0.68673100
C	1.00971500	3.88682800	2.06783200
H	−0.29702300	2.17722800	2.23560500
C	1.64705100	4.51782400	−0.16563400
H	0.84724500	3.29271000	−1.76149200
C	1.72970600	4.73481700	1.21562200
H	1.04663900	4.04846200	3.14781000
H	2.19413500	5.17375500	−0.84715900
C	2.59531200	5.83749000	1.76139900
H	2.41535900	6.78498900	1.23264300
H	2.41852100	5.99924400	2.83321200
H	3.66006800	5.58833600	1.62759300
Ru	−0.86388900	−1.88447300	0.57830100
H	−4.11224400	−3.28668000	0.03103300
C	−3.51984600	−3.91381200	0.71077200
C	−2.46312600	−3.10307900	1.40384200
H	−3.07367900	−4.73393400	0.13242300
H	−4.20575000	−4.35148800	1.45270500
C	−1.21202800	−3.66152100	1.76444200
C	−2.68427300	−1.72597300	1.73726300
C	−0.22535900	−2.92018400	2.50161400
H	−0.99004800	−4.69084600	1.47429900
C	−1.66893200	−0.98216000	2.37032400
H	−3.60657800	−1.22905300	1.43645900
C	−0.43083500	−1.57193900	2.81118400
H	0.72307100	−3.40504400	2.73501500
H	−1.82373900	0.08940500	2.49829000
C	0.60482400	−0.67378600	3.45553300
H	0.70167900	0.18299500	2.76604000
C	1.98203000	−1.31445600	3.59396700
C	0.10523100	−0.14449200	4.80457400
H	2.35971700	−1.69060600	2.63148800
H	2.70491200	−0.57504200	3.96818800
H	1.96774700	−2.15505100	4.30537800
H	0.82163500	0.58001800	5.21977800
H	−0.86726200	0.36091800	4.71084700
H	−0.00750100	−0.96665400	5.52885700
H	−1.64239700	−1.76063400	−0.81268900
N	−4.07445400	1.03206300	−1.42051800
C	−3.72980900	−0.13450400	−2.29727500
H	−2.70557500	0.06432500	−2.64033900
H	−3.67602600	−1.00532800	−1.62997100
C	−4.66208200	−0.39686200	−3.46223600

H	−4.22068700	−1.19735700	−4.07146200
H	−5.65240500	−0.74233500	−3.13735000
H	−4.78276700	0.48137900	−4.11167800
C	−5.34557100	0.87575100	−0.63822700
H	−5.19656600	1.41593200	0.30661600
H	−5.41768500	−0.19137100	−0.38880400
C	−6.61273400	1.35717400	−1.32104100
H	−6.79189500	0.86526500	−2.28447800
H	−7.45930000	1.12018300	−0.66241500
H	−6.61448200	2.44384900	−1.47921600
C	−3.91548600	2.34704100	−2.11624500
H	−2.94901500	2.28454700	−2.63286800
H	−4.70737600	2.43977200	−2.87022500
C	−3.90906600	3.51761100	−1.15130900
H	−3.12745200	3.38640300	−0.38987500
H	−3.69032000	4.43517200	−1.71357000
H	−4.87465400	3.65891300	−0.64649300
H	−3.30598600	1.02691100	−0.70704300

**NAME:**	IEFPCM [Ru]-H + tributylamine-H+
**SINGLE−POINT ENERGY (DFT):**	−2482.72520624 hartree
**CHARGE, MULTIPLICITY:**	1, 1
**STRUCTURE TYPE:**	MINIMUM
**COMMENT:**	…

C	−1.96195800	−0.16476700	0.89541100
H	−1.49565400	−0.15971900	1.89877200
C	−2.75015200	−1.47915200	0.77159800
H	−3.23998100	−1.47941500	−0.21285100
N	−1.77846100	−2.58695500	0.74631700
H	−1.33099300	−2.68756600	1.65750500
H	−2.26566000	−3.46776800	0.57763900
N	−0.92341300	−0.17523100	−0.14532000
C	−3.81291900	−1.62558200	1.83888000
C	−5.16497200	−1.57102100	1.48931200
C	−3.46785800	−1.77489800	3.18717200
C	−6.15602700	−1.66119400	2.46624700
H	−5.44362300	−1.44488900	0.43992500
C	−4.45578300	−1.87139100	4.16523300
H	−2.41715000	−1.80734000	3.48964800
C	−5.80342300	−1.81261200	3.80685900
H	−7.20843300	−1.61190500	2.17861300
H	−4.17254900	−1.98867600	5.21333900
H	−6.57779300	−1.88364500	4.57356400
C	−2.92521000	1.00836300	0.82419400
C	−3.30665300	1.67874800	1.98757600
C	−3.47642900	1.40307300	−0.39785900
C	−4.22245100	2.72939800	1.93413400

H	−2.87875300	1.37659000	2.94713500
C	−4.38556500	2.45557500	−0.45714700
H	−3.16577900	0.89329800	−1.31186100
C	−4.76246300	3.12221900	0.71053700
H	−4.50954000	3.24687100	2.85221600
H	−4.79977600	2.76206800	−1.42028800
H	−5.47534100	3.94854900	0.66462200
S	0.21106600	0.94640300	0.06289700
O	0.62068000	1.11121100	1.47151500
O	1.32201400	0.68170900	−0.88700200
C	−0.46522200	2.50846300	−0.45909700
C	−0.68446000	2.72848100	−1.81783400
C	−0.82004300	3.46453300	0.48685100
C	−1.29240500	3.91096100	−2.22432800
H	−0.38513700	1.98015700	−2.55401900
C	−1.43007300	4.64190400	0.06499600
H	−0.63636700	3.27535000	1.54483700
C	−1.69029700	4.87779000	−1.29032000
H	−1.46960000	4.08350800	−3.28859600
H	−1.72274200	5.38763800	0.80791400
C	−2.41538800	6.12249300	−1.72377400
H	−2.10140900	6.99646500	−1.13544800
H	−2.24887800	6.33818500	−2.78794200
H	−3.50009700	5.99856100	−1.57306300
Ru	−0.31721600	−2.15316800	−0.75247800
H	2.64675600	−4.15400900	−0.54563000
C	1.85291500	−4.67892400	−1.09411500
C	0.88036000	−3.70537100	−1.69512400
H	1.34722300	−5.36644000	−0.40299800
H	2.32651600	−5.27735200	−1.88775400
C	−0.49447500	−4.01803100	−1.83707600
C	1.30781700	−2.41790900	−2.16015100
C	−1.42048400	−3.12876100	−2.48346300
H	−0.86504300	−4.96536000	−1.43881700
C	0.37207000	−1.51689500	−2.70447900
H	2.34257400	−2.10141600	−2.02873900
C	−1.00794500	−1.86552100	−2.92010200
H	−2.46835600	−3.42360900	−2.54788700
H	0.70571900	−0.50470600	−2.93440800
C	−1.93427400	−0.81582700	−3.49700100
H	−1.73126000	0.09723800	−2.91057400
C	−3.41615800	−1.14371200	−3.34605000
C	−1.57771800	−0.52911000	−4.96001700
H	−3.68460400	−1.34794800	−2.29916300
H	−4.02628500	−0.29482300	−3.68721800
H	−3.70155700	−2.02093700	−3.94782600
H	−2.19211300	0.29565700	−5.35055200
H	−0.52083700	−0.24591800	−5.07353400
H	−1.76044700	−1.41729800	−5.58535100
H	0.67260600	−2.11702000	0.50575200
N	3.70447800	0.35835800	0.38616800

C	3.40436800	−0.88018600	1.16998800
H	2.46229800	−0.66524500	1.69418400
H	3.17863200	−1.66083500	0.42986300
C	4.47053600	−1.35388100	2.13924100
H	5.37797100	−1.65679100	1.59364900
H	4.75969400	−0.54125700	2.82543700
C	4.82868100	0.23072700	−0.59562600
H	4.57938200	0.88498900	−1.44302600
H	4.79760300	−0.80288600	−0.96764600
C	6.21598700	0.57078500	−0.07107600
H	6.42057700	0.04109700	0.87076000
H	6.28308400	1.64719000	0.15236400
C	3.74282300	1.59376500	1.22531700
H	2.88739200	1.51344200	1.90855800
H	4.66662400	1.58618500	1.81681500
C	3.59946400	2.85477100	0.38204500
H	2.79668000	2.69331100	−0.35451200
H	4.52356500	3.04607800	−0.18795900
H	2.83552700	0.46750700	−0.19064400
C	3.25381100	4.08232600	1.22516200
H	2.28326800	3.90939600	1.72036900
C	7.28922800	0.20868800	−1.09684500
H	7.24158000	−0.87403800	−1.30206000
H	7.06563300	0.71246800	−2.05242300
C	8.69242600	0.58292800	−0.63490300
H	9.44575900	0.30010600	−1.38420500
H	8.77908600	1.66711400	−0.46301700
H	8.94751200	0.07410000	0.30780800
C	3.95985000	−2.54411400	2.95134500
H	3.64739400	−3.34477800	2.25954300
H	3.05146400	−2.24375300	3.50029400
C	4.99797800	−3.08352900	3.92755400
H	4.60273300	−3.93820400	4.49541900
H	5.90317100	−3.42088800	3.39882900
H	5.30233000	−2.31058700	4.65033300
H	3.09950700	4.93111800	0.54065200
C	4.31110100	4.45021700	2.26183500
H	4.42005200	3.67233000	3.03364300
H	5.29589500	4.59224500	1.78851200
H	4.04897300	5.38567900	2.77730000

2.2. Calculation of substrate−formic acid associates (Table 3):

Optimization of the structures was done on the density functional theory (DFT) level employing the *Gaussian 09* suite [3]. The ωB97XD [4] functional was used with the Def2−SVPD basis set for all atoms [9]. The IEFPCM solvation model was applied using acetonitrile with the default value of dielectric constant.

**NAME:**	1-Me-3,4-DHIQ
**SINGLE−POINT ENERGY (DFT):**	−442.00581411 hartree
**CHARGE, MULTIPLICITY:**	0, 1
**STRUCTURE TYPE:**	MINIMUM
**COMMENT:**	…

H	−3.84604000	−0.48575500	−0.04537200
C	−2.78693700	−0.22421100	−0.03132800
C	−1.82625100	−1.22210900	0.12891800
C	−2.39469100	1.10697900	−0.17230300
C	−0.46891300	−0.90330400	0.14211400
H	−2.13316600	−2.26338000	0.24341000
C	−1.04131400	1.43549300	−0.15210700
H	−3.14341300	1.88995400	−0.29673900
C	−0.07011500	0.43842800	0.00038200
C	0.61355900	−1.92859000	0.34124900
H	−0.74456000	2.47842800	−0.26204200
C	1.38950600	0.74388500	0.02813100
C	1.86189800	−1.51633600	−0.42726300
H	0.85079100	−1.99849700	1.41671800
H	0.27576900	−2.92366500	0.02323900
N	2.28466500	−0.15017200	−0.15589100
C	1.82134800	2.16395000	0.26691700
H	1.68018500	−1.60709700	−1.51298400
H	2.69610100	−2.18927600	−0.19173500
H	1.35703800	2.56921900	1.17703400
H	2.91083100	2.20687600	0.36125500
H	1.51525700	2.80927800	−0.56980100

**NAME:**	1-Me-3,4-DHIQ + H^+
**SINGLE−POINT ENERGY (DFT):**	−442.46874584 hartree
**CHARGE, MULTIPLICITY:**	1, 1
**STRUCTURE TYPE:**	MINIMUM
**COMMENT:**	…

C	−2.80635800	−0.25199400	−0.03263000
C	−1.83676600	−1.24276200	0.11089800
C	−2.44007500	1.08997600	−0.15555400
C	−0.48499800	−0.91111800	0.12067500
H	−2.13307800	−2.28698900	0.21476400
C	−1.09759800	1.43982100	−0.13329100
C	−0.11517400	0.44499400	−0.00275500
C	0.59343000	−1.94015200	0.32769000
H	−0.81568000	2.48703000	−0.23173600
C	1.30225000	0.79368700	0.03529200
C	1.86036000	−1.54174800	−0.40441400
H	0.79532300	−2.03617800	1.40627600
N	2.18669500	−0.14364800	−0.11659800
C	1.76295100	2.19304800	0.23685800
H	1.73868700	−1.63829000	−1.49315900
H	2.71408800	−2.15221200	−0.09837400
H	1.28456200	2.62028000	1.12661000
H	2.85057000	2.24891700	0.34189300
H	1.46054400	2.79913100	−0.62876200
H	−3.20207000	1.86041100	−0.26736200
H	−3.86106000	−0.52873700	−0.04731700
H	3.16834500	0.11312700	−0.09577900
H	0.26476700	−2.92346000	−0.02747900

**NAME:**	1-Me-3,4-DHIQ + FA
**SINGLE−POINT ENERGY (DFT):**	−631.61161096 hartree
**CHARGE, MULTIPLICITY:**	0, 1
**STRUCTURE TYPE:**	MINIMUM
**COMMENT:**	…

C	4.03023600	−0.06909500	−0.39974200
C	3.21606800	1.03488000	−0.15034100
C	3.48761600	−1.35442300	−0.40572300
C	1.85236900	0.86886300	0.08524900
H	3.64382800	2.03870900	−0.14232100
C	2.12975800	−1.53251700	−0.15980000
C	1.30392500	−0.42702700	0.08056700
C	0.92945000	2.01722300	0.39126200
H	1.71297400	−2.53917400	−0.16450400

C	−0.14724800	−0.57630300	0.34980300
C	−0.45751200	1.72227400	−0.15614700
H	0.87278600	2.15560900	1.48386300
N	−0.95485500	0.41573600	0.25867900
C	−0.68843400	−1.92216100	0.72911800
H	−0.44535200	1.74552400	−1.25884800
H	−1.17928600	2.47926400	0.17258600
H	−0.13390300	−2.33412000	1.58277000
H	−1.74963100	−1.85263100	0.98492600
H	−0.57483800	−2.62683400	−0.10684100
H	4.12291500	−2.21841000	−0.60157100
H	5.09463100	0.07495600	−0.58957700
H	−2.45557300	0.30672700	0.47121000
H	1.31466000	2.95315100	−0.03210100
C	−4.04293700	−0.15272500	−0.54776800
O	−3.50210100	0.25287500	0.56641000
O	−5.23601800	−0.30124000	−0.70987900
H	−3.31401900	−0.35992300	−1.36145700

**NAME:**	6-MeO-1-Me-3,4-DHIQ
**SINGLE−POINT ENERGY (DFT):**	−556.42183015 hartree
**CHARGE, MULTIPLICITY:**	0, 1
**STRUCTURE TYPE:**	MINIMUM
**COMMENT:**	…

C	1.98795700	−0.21063600	−0.02564100
C	1.01547200	−1.21212900	−0.16241800
C	1.59609400	1.12592500	0.11302700
C	−0.33317300	−0.89185400	−0.15260800
H	1.33983900	−2.24758600	−0.27460100
C	0.23834300	1.44030600	0.11151300
C	−0.73983200	0.45167000	−0.01296200
C	−1.41633600	−1.92013900	−0.33216300
H	−0.04911100	2.48614700	0.21862800
C	−2.19526700	0.75280600	−0.01583800
C	−2.65562500	−1.50593600	0.45106700
H	−1.66690200	−1.99160400	−1.40434800
N	−3.08832600	−0.14286700	0.17976300
C	−2.63447300	2.17271800	−0.24504500
H	−2.45697600	−1.59163100	1.53440500
H	−3.48942800	−2.18472900	0.23110400
H	−2.19325100	2.57789200	−1.16665000
H	−3.72601100	2.21508100	−0.31230700
H	−2.30819300	2.81888900	0.58333200
H	−1.07387900	−2.91425000	−0.01645000
O	3.27158000	−0.62388600	−0.03907800
C	4.30216700	0.33105100	0.10633700
H	4.22417200	0.85272800	1.07218500

H	5.24422800	−0.22223300	0.06852400
H	4.27971100	1.06758400	−0.71086100
H	2.32948100	1.92219000	0.21971800

**NAME:**	6-MeO-1-Me-3,4-DHIQ + H^+
**SINGLE−POINT ENERGY (DFT):**	−556.88822873 hartree
**CHARGE, MULTIPLICITY:**	1, 1
**STRUCTURE TYPE:**	MINIMUM

C	2.01696700	−0.21258400	−0.02080100
C	1.03916300	−1.21683000	−0.13889300
C	1.63516300	1.13581000	0.09099000
C	−0.30297500	−0.89530200	−0.13037700
H	1.36343600	−2.25269800	−0.23675300
C	0.28923900	1.45809000	0.08348700
C	−0.69545800	0.46219800	−0.01474500
C	−1.37442200	−1.93514500	−0.32151900
H	0.00637500	2.50595400	0.17346200
C	−2.10435400	0.79407100	−0.02477100
C	−2.63709800	−1.54523300	0.42379400
H	−1.58757400	−2.03005200	−1.39793700
N	−2.98337300	−0.15402400	0.13378700
C	−2.58792000	2.19121000	−0.20353200
H	−2.49845700	−1.63748300	1.51117500
H	−3.48573400	−2.16979300	0.13186700
H	−2.14674100	2.62928600	−1.10699500
H	−3.67911000	2.23361200	−0.27302700
H	−2.26699800	2.79789000	0.65449900
H	−3.96647000	0.09442000	0.12989000
H	−1.03379300	−2.91616300	0.02839600
O	3.28359300	−0.62863900	−0.02564200
C	4.33580000	0.31437500	0.10161800
H	4.26537800	0.84862700	1.05973300
H	5.26446400	−0.26007300	0.07058800
H	4.32002400	1.03317800	−0.72970900
H	2.37544600	1.92661800	0.18193000

**NAME:**	6-MeO-1-Me-3,4-DHIQ + FA
**SINGLE−POINT ENERGY (DFT):**	−746.03310727 hartree
**CHARGE, MULTIPLICITY:**	0, 1
**STRUCTURE TYPE:**	MINIMUM
**COMMENT:**	…

C	−3.21078800	0.05276100	−0.05206300
C	−2.35046000	1.15076900	−0.22257300
C	−2.68028800	−1.22965600	0.15854600
C	−0.97917900	0.98494700	−0.17185200
H	−2.78670900	2.13504600	−0.39339100
C	−1.30347900	−1.39420200	0.19507200
C	−0.43749700	−0.30409500	0.04248900
C	−0.02278300	2.12247100	−0.41198800
H	−0.90233100	−2.39328500	0.36016200
C	1.01166100	−0.46943800	0.07942600
C	1.25138400	1.91883200	0.38696200
H	0.21395200	2.16433000	−1.48734700
N	1.77414700	0.56895200	0.20376300
C	1.64743700	−1.81552300	−0.00995600
H	1.06599200	2.06692000	1.46201900
H	2.03339400	2.62115000	0.08259500
H	1.22539600	−2.37567400	−0.85348800
H	2.73413600	−1.72901700	−0.12931100
H	1.42452800	−2.37918400	0.90746700
H	2.83595900	0.46820100	0.24907300
H	−0.47908200	3.08239800	−0.14485500
O	−4.52063700	0.32172100	−0.10649600
C	−5.45940200	−0.72633000	0.05885700
H	−5.35264800	−1.19624300	1.04727900
H	−6.44676500	−0.26543600	−0.02195300
H	−5.34400600	−1.48563300	−0.72817400
H	−3.32593100	−2.09418600	0.29217400
C	5.20235500	−0.25511200	−0.01075600
O	4.40464400	0.61587800	0.43672600
O	4.91428700	−1.28181600	−0.64507400
H	6.28496600	−0.06086200	0.19717800

**NAME:**	7-MeO-1-Me-3,4-DHIQ
**SINGLE−POINT ENERGY (DFT):**	−556.41975757 hartree
**CHARGE, MULTIPLICITY:**	0, 1
**STRUCTURE TYPE:**	MINIMUM
**COMMENT:**	…

C	1.51519600	−1.79463700	−0.07716300
C	0.17014700	−2.12760700	−0.15889200
C	1.90115800	−0.45025300	0.01357500
C	−0.81941400	−1.14186000	−0.14215500
H	−0.11825000	−3.17766300	−0.23362000
C	0.92561800	0.54755300	0.02023800
C	−0.43216300	0.20194400	−0.05378300
C	−2.28984600	−1.43980900	−0.25530100
H	1.20990400	1.59462300	0.08899600
C	−1.50312900	1.24115300	−0.05292200
C	−3.08138800	−0.40461800	0.53195000
H	−2.59160300	−1.40381000	−1.31641400
N	−2.72562700	0.96838600	0.20301300
C	−1.12664600	2.66550600	−0.35307800
H	−2.91125900	−0.54872200	1.61394400
H	−4.15811600	−0.53206300	0.36313700
H	−0.58193900	2.73602300	−1.30533300
H	−2.02990200	3.28135100	−0.40095200
H	−0.46770500	3.06825700	0.43030400
H	−2.51498200	−2.45135200	0.10741000
O	3.23136700	−0.21151700	0.09158100
C	3.68248900	1.12235100	0.18553900
H	4.77330400	1.07600700	0.24448900
H	3.39009100	1.70527300	−0.70169800
H	3.29042500	1.61245300	1.09010800
H	2.28635200	−2.56526600	−0.08216200

**NAME:**	7-MeO-1-Me-3,4-DHIQ + H^+
**SINGLE−POINT ENERGY (DFT):**	−556.88263965 hartree
**CHARGE, MULTIPLICITY:**	1, 1
**STRUCTURE TYPE:**	MINIMUM
**COMMENT:**	…

C	1.55174400	−1.78442100	−0.07104200
C	0.21251100	−2.13168100	−0.14444600
C	1.94020600	−0.43659800	0.01386700
C	−0.78530000	−1.15553700	−0.12112600
H	−0.06610900	−3.18325100	−0.21771400
C	0.96345000	0.55459300	0.02132900
C	−0.39464500	0.19129500	−0.03957400
C	−2.24703900	−1.48914300	−0.25196900
H	1.24107300	1.60289900	0.08847500
C	−1.43034700	1.22236100	−0.05979800
C	−3.08697100	−0.47854800	0.50345100
H	−2.52726300	−1.49234500	−1.31751000
N	−2.66332100	0.87982800	0.15626200
C	−1.11333800	2.65107000	−0.32246800
H	−2.96718900	−0.59593800	1.59050500

H	−4.14919000	−0.56809100	0.26209000
H	−0.55106300	2.74041000	−1.26048400
H	−2.01840900	3.26320100	−0.37640000
H	−0.47650200	3.03490700	0.48642900
H	−3.37450900	1.60396800	0.14272900
H	−2.45251000	−2.49198300	0.13920000
O	3.26324300	−0.20101400	0.08452700
C	3.72027800	1.13434400	0.17025400
H	4.81056700	1.08254700	0.22309100
H	3.42393600	1.71091500	−0.71916100
H	3.33423800	1.62623200	1.07586400
H	2.32694600	−2.55057300	−0.07802400

**NAME:**	7-MeO-1-Me-3,4-DHIQ + FA
**SINGLE−POINT ENERGY (DFT):**	−746.02889157 hartree
**CHARGE, MULTIPLICITY:**	0, 1
**STRUCTURE TYPE:**	MINIMUM
**COMMENT:**	…

C	−3.24054800	1.14724000	−0.24760800
C	−2.16937700	2.02350100	−0.32887100
C	−3.02610400	−0.22416200	−0.03825000
C	−0.85594300	1.56840300	−0.19353500
H	−2.35515300	3.08495100	−0.49681500
C	−1.72307700	−0.70102500	0.08441200
C	−0.64547900	0.19690500	0.01111500
C	0.33963200	2.47451500	−0.32060700
H	−1.53479900	−1.75865800	0.24791500
C	0.73705600	−0.29256500	0.12157800
C	1.47023200	1.97111900	0.55631800
H	0.66696800	2.50297300	−1.37266200
N	1.69330000	0.54380600	0.34573100
C	1.06262900	−1.74145000	−0.00573700
H	1.23814000	2.12132700	1.62196700
H	2.41234500	2.48531100	0.34263300
H	0.55057400	−2.16962900	−0.87617400
H	2.14278300	−1.88895800	−0.10818500
H	0.69996200	−2.26971100	0.88801200
H	2.70895000	0.20103200	0.44289300
H	0.08319700	3.50176900	−0.03627200
O	−4.12695700	−1.00007500	0.02935800
C	−3.97224700	−2.38909100	0.23811100
H	−4.97957200	−2.81255600	0.26149100
H	−3.40188700	−2.85189400	−0.58163700
H	−3.47033000	−2.59157600	1.19656500
H	−4.26471500	1.50785100	−0.34536900
C	4.80085100	−0.88980600	−0.08349900

O	4.21273500	−0.09688700	0.70705300
O	4.30148400	−1.48739600	−1.04814300
H	5.88659700	−1.05553100	0.12880400

**NAME:**	6,7-diMeO-1-Me-3,4-DHIQ
**SINGLE−POINT ENERGY (DFT):**	−670.82408541 hartree
**CHARGE, MULTIPLICITY:**	0, 1
**STRUCTURE TYPE:**	MINIMUM
**COMMENT:**	…

C	1.47462800	−0.74587900	0.04318100
C	0.35447000	−1.57639300	0.08033400
C	1.29956400	0.64771400	−0.04713700
C	−0.93296000	−1.05251100	0.01424400
H	0.51233900	−2.65301100	0.15825500
C	0.01344200	1.17013500	−0.13587400
C	−1.10863600	0.33622400	−0.10638500
C	−2.17001900	−1.90888600	0.01078400
H	−0.08665000	2.25227600	−0.21452900
C	−2.49731400	0.86578100	−0.20108300
C	−3.31322000	−1.15801800	0.68040100
H	−2.44496600	−2.14134000	−1.03230800
N	−3.51998200	0.17994200	0.14384400
C	−2.69877900	2.26566600	−0.71043300
H	−3.11468400	−1.06194400	1.76275100
H	−4.25160500	−1.71788900	0.58084800
H	−2.19335700	2.41065900	−1.67555500
H	−3.76871800	2.46560200	−0.82293100
H	−2.27631300	2.99835800	−0.00682500
H	−1.98911000	−2.86658100	0.51565300
O	2.70868100	−1.31096800	0.14309200
O	2.36426300	1.50192500	−0.08216900
C	3.56780800	−1.15890900	−0.98001400
H	3.77843500	−0.10018600	−1.18088000
H	4.49842600	−1.67966700	−0.73360000
H	3.11340000	−1.61996800	−1.86956000
C	3.08542100	1.63085000	1.13505300
H	3.88324700	2.35811100	0.95308100
H	2.42411000	2.00262200	1.93218400
H	3.52532200	0.67105000	1.44070000

**NAME:**	6,7-diMeO-1-Me-3,4-DHIQ + H^+
**SINGLE−POINT ENERGY (DFT):**	−671.28902884 hartree
**CHARGE, MULTIPLICITY:**	1, 1
**STRUCTURE TYPE:**	MINIMUM
**COMMENT:**	…

C	1.49340000	−0.86245200	−0.06765300
C	0.33118700	−1.64972500	0.00300400
C	1.36402800	0.54568100	−0.15628300
C	−0.93050900	−1.08798400	−0.01646200
H	0.45533200	−2.73060700	0.06676500
C	0.09869500	1.10668900	−0.20536400
C	−1.05673700	0.31552400	−0.12656300
C	−2.18222100	−1.92391400	−0.02312500
H	0.03521200	2.19137200	−0.28356000
C	−2.37812200	0.90669700	−0.18317500
C	−3.31803900	−1.18385500	0.65725500
H	−2.45336000	−2.14884000	−1.06680400
N	−3.41068500	0.18208100	0.13933300
C	−2.59276100	2.32233600	−0.59041400
H	−3.15423500	−1.11805900	1.74302500
H	−4.28039700	−1.67274300	0.48362100
H	−2.11118000	2.51048200	−1.55776300
H	−3.65698700	2.56658800	−0.65786000
H	−2.12597300	2.98554000	0.15082900
H	−2.01723200	−2.88207600	0.48198500
O	2.64118700	−1.53876200	−0.02289400
O	2.45947600	1.35581300	−0.20033400
C	3.88012400	−1.01336800	−0.49116000
H	4.31516400	−0.31461700	0.23190300
H	4.53638000	−1.88099000	−0.60300200
H	3.75145200	−0.51558200	−1.45901400
C	2.83634300	1.92033800	1.04762500
H	3.74364400	2.50587300	0.86930100
H	2.04345400	2.57715200	1.43498200
H	3.04639300	1.13201800	1.78704300
H	−4.33051000	0.60771900	0.10292800

**NAME:**	6,7-diMeO-1-Me-3,4-DHIQ + FA
**SINGLE−POINT ENERGY (DFT):**	−860.43412027 hartree
**CHARGE, MULTIPLICITY:**	0, 1
**STRUCTURE TYPE:**	MINIMUM
**COMMENT:**	…

C	−2.70629400	0.65163600	−0.12974900
C	−1.72095500	1.64662000	−0.09132500
C	−2.31643500	−0.70569400	−0.08184000
C	−0.37362900	1.32926800	−0.02915200
H	−2.04364200	2.68743700	−0.12657700
C	−0.96722400	−1.02482100	−0.05079000
C	0.01352000	−0.02620600	−0.02078100
C	0.70504600	2.37902800	−0.06069100
H	−0.70207600	−2.08111900	−0.02043300
C	1.43686800	−0.35792500	0.00046100
C	1.92196000	1.90333700	0.71131600
H	0.98052900	2.57750800	−1.10885400
N	2.30146600	0.55281600	0.30798600
C	1.92141500	−1.73199700	−0.31404500
H	1.71694200	1.88676800	1.79287900
H	2.78636000	2.55296800	0.54338200
H	1.45105600	−2.09557200	−1.23607200
H	3.01212000	−1.74783500	−0.42470100
H	1.62520900	−2.41177600	0.49790400
H	0.34423000	3.32320100	0.36326600
O	−3.97986300	1.07291300	−0.17239700
O	−3.24247400	−1.70735400	−0.06388600
C	−4.97231900	0.35160600	−0.89644000
H	−5.43097400	−0.42832500	−0.27767000
H	−5.72997000	1.08904200	−1.17825200
H	−4.54320400	−0.09997400	−1.79943400
C	−3.74898000	−2.01374300	1.22730200
H	−4.50869300	−2.79088400	1.09620000
H	−2.94540800	−2.38753600	1.87930300
H	−4.20712900	−1.12677900	1.69174200
H	3.34769800	0.32999500	0.34196700
C	5.61115500	−0.54267800	−0.11592400
O	4.90874800	0.26770200	0.55217400
O	5.21383900	−1.35738900	−0.96267100
H	6.70992000	−0.50439900	0.09363800

**NAME:**	1-Ph-3,4-DHIQ
**SINGLE−POINT ENERGY (DFT):**	−633.54720922 hartree
**CHARGE, MULTIPLICITY:**	0, 1
**STRUCTURE TYPE:**	MINIMUM
**COMMENT:**	…

H	4.11261900	−2.54962100	−0.25349000
C	3.24599600	−1.88789500	−0.21645000
C	3.39850900	−0.56751700	0.20562300
C	1.99090400	−2.35887200	−0.60205800
C	2.30130900	0.29104800	0.25280200

H	4.38349600	−0.19475800	0.49224200
C	0.88682400	−1.51194800	−0.54754900
H	1.87089600	−3.38622400	−0.94720600
C	1.03001500	−0.18941500	−0.11120600
C	2.39729600	1.73254800	0.67124000
H	−0.09178500	−1.88094500	−0.85435700
C	−0.11129500	0.76790400	−0.05450000
C	1.40790400	2.55668600	−0.14286100
H	2.15405800	1.82433500	1.74355800
H	3.41810100	2.11320200	0.53829400
N	0.05116000	2.03685400	−0.07416500
H	1.71639600	2.57340100	−1.20369600
H	1.39635400	3.59886700	0.19992900
C	−1.50863600	0.24455200	0.01290000
C	−2.50604900	0.82213200	−0.78114800
C	−1.85166100	−0.79136800	0.89075600
C	−3.82089300	0.36867500	−0.70423900
H	−2.23743000	1.62858800	−1.46414400
C	−3.16958600	−1.23554200	0.97755700
H	−1.08410900	−1.24417300	1.51984500
C	−4.15655300	−0.65973500	0.17731300
H	−4.58766700	0.81878900	−1.33645500
H	−3.42681100	−2.03399600	1.67488600
H	−5.18674200	−1.01296800	0.24067800

**NAME:**	1Ph-3,4-DHIQ + H^+
**SINGLE−POINT ENERGY (DFT):**	−634.00764971 hartree
**CHARGE, MULTIPLICITY:**	1, 1
**STRUCTURE TYPE:**	MINIMUM
**COMMENT:**	…

H	4.11259100	−2.55709500	−0.24952200
C	3.24280600	−1.90025000	−0.21374300
C	3.40210200	−0.57020500	0.17459700
C	1.98634700	−2.39361600	−0.56926300
C	2.30773900	0.28698300	0.21884900
H	4.39044800	−0.19237900	0.43753500
C	0.88064700	−1.55652800	−0.51798100
H	1.87067900	−3.42844400	−0.88911100
C	1.03245200	−0.21946900	−0.11548900
C	2.42412400	1.72818800	0.63724800
H	−0.09888200	−1.93175500	−0.81019400
C	−0.11396700	0.68001600	−0.04531900
C	1.42003600	2.57459600	−0.12353800
H	2.24105500	1.81201500	1.72012600
H	3.43537100	2.10602000	0.44954200
N	0.09339600	1.96519200	−0.02198100
H	1.68139600	2.64313100	−1.18961300

H	1.35060500	3.58732400	0.28183700
C	−1.50465300	0.19788400	0.00694800
C	−2.48481300	0.80161300	−0.79411100
C	−1.85665600	−0.83571700	0.88696400
C	−3.80411600	0.36770200	−0.71743600
H	−2.21146500	1.58501300	−1.50267600
C	−3.18096600	−1.25097300	0.96926700
H	−1.09856100	−1.29406500	1.52153100
C	−4.15321700	−0.65367100	0.16601800
H	−4.56096900	0.82538300	−1.35432200
H	−3.45562700	−2.04322000	1.66550900
H	−5.18914000	−0.98817200	0.22800000
H	−0.70846000	2.58058000	0.06716200

**NAME:**	1-Ph-3,4-DHIQ + FA
**SINGLE−POINT ENERGY (DFT):**	−823.15352027 hartree
**CHARGE, MULTIPLICITY:**	0, 1
**STRUCTURE TYPE:**	MINIMUM
**COMMENT:**	…

H	5.48180600	0.59397400	0.54777600
C	4.40603800	0.45765900	0.42991300
C	3.91393000	−0.72497900	−0.12136700
C	3.52732400	1.45914800	0.84387600
C	2.54204600	−0.91803200	−0.27101500
H	4.60367200	−1.51131900	−0.43201900
C	2.15600100	1.28285300	0.68688100
H	3.91089800	2.37713900	1.28937700
C	1.65638000	0.10267300	0.12240600
C	1.94479500	−2.17547100	−0.84258300
H	1.46830900	2.06182000	1.01475300
C	0.20345500	−0.13277000	−0.06537200
C	0.61945300	−2.45935100	−0.15227500
H	1.77584200	−2.04423800	−1.92440000
H	2.62971100	−3.02375500	−0.72218100
N	−0.27861700	−1.31347100	−0.20360200
H	0.78965300	−2.71432300	0.90752800
H	0.10917200	−3.31383800	−0.61232800
C	−0.74217500	1.01721300	−0.09708600
C	−1.93719200	0.95412600	0.62937700
C	−0.47048000	2.14676600	−0.87927400
C	−2.84684700	2.00758700	0.57442800
H	−2.14767600	0.07481800	1.23965300
C	−1.38671100	3.19368400	−0.94079500
H	0.45691300	2.20057500	−1.45058200
C	−2.57487000	3.12709600	−0.21233600
H	−3.77261800	1.95356100	1.14855300
H	−1.17199800	4.06480600	−1.56087200

H	−3.29015200	3.94935900	−0.25661700
H	−1.77516100	−1.65729100	−0.51944400
C	−3.51273100	−2.27560300	0.14441200
O	−2.72235500	−1.94868900	−0.84011000
O	−3.22720700	−2.25175700	1.32455100
H	−4.51204500	−2.58901600	−0.21466400

2.3 Transition state of hydrogenation of 1-phenyl-DHIQ with [RuH(η^6-mesitylene)(S,S)-TsDPEN]:

The geometry optimization and frequency analysis was performed as described in Section 2.1

**NAME:**	1-Ph-3,4-DHIQ + Ru cat.
**SINGLE−POINT ENERGY (DFT):**	−2549.17570815 hartree
**CHARGE, MULTIPLICITY:**	1, 1
**STRUCTURE TYPE:**	TRANSITION STATE
**COMMENT:**	…

Ru	1.01270800	−0.26119900	1.34069300
S	−0.87694800	−1.64827600	−0.92990900
O	−0.10872600	−2.83293900	−0.53376500
O	−0.47271200	−1.02641300	−2.22051700
N	0.34619100	1.68744100	0.83339600
N	−0.85886500	−0.59068100	0.28835200
C	−1.12204100	1.74660700	0.91928800
C	−1.67932700	0.61010700	0.05243200
C	2.71102700	−1.40784800	2.28254200
C	2.62530300	−0.09588000	2.82324700
C	1.40218500	0.37811100	3.37411300
C	0.26761500	−0.48662700	3.41222200
C	0.32730900	−1.76930700	2.81763100
C	1.55017100	−2.21539000	2.24207700
H	−1.37793600	1.54269700	1.96847000
H	−1.58912800	0.92956600	−1.00246500
H	0.74965000	2.42803200	1.40907300
H	0.63154800	1.90448000	−0.12372100
H	3.50097500	0.55478300	2.80178000
H	1.58666900	−3.19033900	1.75644200
H	1.98363400	−0.09654700	−0.10061700
C	−2.56692100	−2.14847700	−1.19450800
C	−3.14846200	−1.96412400	−2.44477500
C	−3.29298500	−2.71779900	−0.14858300
C	−4.47700100	−2.33513800	−2.64086600
H	−2.56612200	−1.51714800	−3.25056200
C	−4.61754600	−3.07628700	−0.35650500
H	−2.83634500	−2.85166700	0.83146100
C	−5.23576400	−2.88118500	−1.60068700
H	−4.93530400	−2.18272300	−3.62103400
H	−5.19032400	−3.50482000	0.46932300

C	−3.15712800	0.44013000	0.37035200
C	−4.13231100	0.72979100	−0.58400700
C	−3.56066200	0.01545100	1.64012300
C	−5.48661700	0.57776900	−0.28548200
H	−3.82864100	1.06183000	−1.58021700
C	−4.91039900	−0.13141300	1.94643400
H	−2.80535900	−0.22104600	2.39256800
C	−5.87926000	0.14530600	0.98005400
H	−6.23764700	0.79354300	−1.04856000
H	−5.20845900	−0.47053800	2.94104100
H	−6.93910700	0.02126300	1.21297400
C	−1.68011700	3.10177600	0.54134400
C	−2.43664600	3.83124500	1.46166000
C	−1.45231900	3.64204800	−0.73052400
C	−2.95803400	5.07946900	1.12048100
H	−2.62313400	3.41531100	2.45509600
C	−1.96433000	4.89172700	−1.07089700
H	−0.86866700	3.08928400	−1.47216700
C	−2.72127900	5.61288800	−0.14565800
H	−3.55078800	5.63766800	1.84812700
H	−1.77483000	5.30269100	−2.06475500
H	−3.12771100	6.59052200	−0.41308700
C	−6.69046500	−3.21247500	−1.78974400
H	−7.31831200	−2.40177200	−1.38558600
H	−6.94283100	−3.33318000	−2.85199800
H	−6.96480300	−4.13445900	−1.25754000
C	4.02157400	−1.95620500	1.81190000
H	4.47912500	−2.49159900	2.65856300
H	3.89324200	−2.67378800	0.99333700
H	4.71284600	−1.16611900	1.50093400
C	1.31490700	1.76496100	3.94232600
H	0.30892000	2.19144900	3.82373900
H	1.53049300	1.72655200	5.02054700
H	2.05127600	2.43574800	3.47891600
C	−0.86718300	−2.66992100	2.78032300
H	−0.78882900	−3.40144500	3.59884800
H	−1.80279800	−2.11216000	2.90431700
H	−0.89146600	−3.21457000	1.82825000
H	−0.67039700	−0.12880200	3.83986900
H	7.94190200	−0.64304600	−0.02521600
C	6.89137700	−0.52536700	−0.29787800
C	6.23327400	−1.53577700	−0.98818000
C	6.19554000	0.63208000	0.05137200
C	4.88538600	−1.41550300	−1.34115900
H	6.76597100	−2.45253100	−1.25172400
C	4.86093700	0.77149000	−0.30764300
H	6.69159800	1.43198300	0.60359200
C	4.18645500	−0.23667000	−1.01215700
C	4.19723000	−2.54396200	−2.06310900
H	4.35122000	1.68412100	−0.01642200
C	2.76260500	−0.07947800	−1.48512600

C	2.70319700	−2.46753500	−1.83897100
H	4.41687700	−2.47772700	−3.14229300
H	4.58948100	−3.50950700	−1.71409200
N	2.25495900	−1.12773900	−2.14024100
H	2.43490500	−2.70478400	−0.79212700
H	2.15264000	−3.16547300	−2.47968500
C	2.31363600	1.26275000	−2.01461100
C	2.36448900	2.44763000	−1.26540100
C	1.87065600	1.34029400	−3.34227600
C	1.98051500	3.66577800	−1.81934700
H	2.67428400	2.42635800	−0.22184800
C	1.47749100	2.55824400	−3.89477100
H	1.85091700	0.44269400	−3.96055300
C	1.53189100	3.72617200	−3.13825700
H	2.01773200	4.56882700	−1.20747900
H	1.13349000	2.59012400	−4.93029900
H	1.22419700	4.67951500	−3.57183900
H	1.26504000	−1.04317000	−2.41239000

References

1. Pecháček, J.; Václavík, J.; Přech, J.; Šot, P.; Januščák, J.; Vilhanová, B.; Vavřík, J.; Kuzma, M.; Kačer, P. Asymmetric Transfer Hydrogenation of Imines Catalyzed by a Noyori-Type Ru(II) Complex – A Parametric Study. *Tetrahedron: Asymmetry* **2013**, *24*, 233–239.
2. Václavík, J.; Pecháček, J.; Vilhanová, B.; Šot, P.; Januščák, J.; Matoušek, V.; Přech, J.; Bártová, S.; Kuzma, M.; Kačer, P. Molecular Structure Effects in the Asymmetric Transfer Hydrogenation of Functionalized Dihydroisoquinolines on (*S*,*S*)-[RuCl(η^6-*p*-cymene)TsDPEN]. *Catal. Lett.* **2013**, *143*, 555–562.
3. Přech, J.; Václavík, J.; Šot, P.; Pecháček, J.; Vilhanová, B.; Januščák, J.; Syslová, K.; Pažout, R.; Maixner, J.; Zápal, J.; *et al.* Asymmetric transfer hydrogenation of 1-phenyl dihydroisoquinolines using Ru(II) diamine catalysts. *Catal. Commun.* **2013**, *36*, 67–70.
4. Přech, J.; Matoušek, V.; Václavík, J.; Pecháček, J.; Syslová, K.; Šot, P.; Januščák, J.; Vilhanová, B.; Kuzma, M.; Kačer, P. Determination of enantiomeric composition of substituted tetrahydroisoquinolines based on derivatization with menthyl chloroformate. *Am. J. Anal. Chem.* **2013**, *4*, 125–133.
5. Frisch, M.J.; Trucks, G.W.; Schlegel, H.B.; Scuseria, G.E.; Robb, M.A.; Cheeseman, J.R.; Scalmani, G.; Barone, V.; Mennucci, B.; Petersson, G.A.; *et al. Gaussian 09*, Revision C.01; Gaussian, Inc.: Wallingford, CT, USA, 2013.
6. Chai, J.-D.; Head−Gordon, M. Long−range corrected hybrid density functionals with damped atom−atom dispersion corrections. *Phys. Chem. Chem. Phys.* **2008**, *10*, 6615–6620.
7. Weigend, F.; Ahlrichs, R. Balanced basis sets of split valence, triple zeta valence and quadruple zeta valence quality for H to Rn: Design and assessment of accuracy. *Phys. Chem. Chem. Phys.* **2005**, *7*, 3297–3305.

8. Andrae, D.; Häußermann, U.; Dolg, M.; Stoll, H.; Preuß, H. Energy−adjusted *ab initio* pseudopotentials for the second and third row transition elements. *Theor. Chem. Acc.* **1990**, *77*, 123–141.
9. Wu, J.; Wang, F.; Ma, Y.; Cui, X.; Cun, L.; Zhu, J.; Deng, J.; Yu, B. Asymmetric transfer hydrogenation of imines and iminiums catalyzed by a water-soluble catalyst in water. *Chem. Commun.* **2006**, 1766–1768.

© 2014 by the authors; licensee MDPI, Basel, Switzerland. This article is an open access article distributed under the terms and conditions of the Creative Commons Attribution license (http://creativecommons.org/licenses/by/3.0/).
